# Barrier model selection alters apparent diclofenac permeation parameters: a controlled *in vitro* comparison of Strat-M^®^ and a HaCaT-based epidermal construct

**DOI:** 10.3389/fphar.2026.1934405

**Published:** 2026-10-07

**Authors:** Filip Dugonik, Urška Jeršič, Lejla Kač, Lidija Gradišnik, Tanja Zidarič, Uroš Maver, Tina Maver

**Affiliations:** 1 Institute of Biomedical Sciences, Faculty of Medicine, University of Maribor, Maribor, Slovenia; 2 Department of Pharmacology, Faculty of Medicine, University of Maribor, Maribor, Slovenia

**Keywords:** barrier model, diclofenac, Franz diffusion cell, HaCaT, *In vitro* permeation testing, permeation profile, Strat-M^®^, topical gel

## Abstract

**Introduction:**

Synthetic membranes and keratinocyte-based constructs are both used as accessible alternatives to *ex vivo* skin in *in vitro* permeation testing (IVPT), but they have not been compared directly for the same marketed products under identical conditions. This pilot study directly compared the synthetic Strat-M® membrane and a HaCaT construct under identical IVPT conditions using Voltaren® and Olfen®, two marketed, nominally dose-equivalent diclofenac gels. The aim was to determine whether the two barrier models yielded consistent apparent permeation parameters and led to the same comparative interpretation of the two formulations.

**Methods:**

Permeation studies were performed using Franz diffusion cells in an internally controlled experimental design. Both formulations were tested in triplicate under identical conditions, including receptor medium, temperature, sampling protocol, and analytical method. Steady-state flux (Jss), permeability coefficient (Kp), lag time (*t*lag), and cumulative permeation profiles were determined and compared between barrier models.

**Results:**

Barrier model selection affected the apparent permeation behavior of diclofenac. Strat-M® detected a statistically significant difference in steady-state flux between Voltaren® and Olfen®, whereas no statistically significant between-formulation difference was detected with the HaCaT construct under the same experimental conditions. Lag time was the parameter most strongly influenced by barrier selection and was consistently shorter with the HaCaT construct than with Strat-M®.

**Discussion:**

Under identical IVPT conditions, Strat-M® and the HaCaT-based epidermal construct generated different apparent permeation profiles and yielded different comparative assessments of Voltaren® and Olfen®. This model-dependent divergence underscores the need to justify barrier selection explicitly and to consider its limitations when interpreting comparative IVPT data.

## 1 Introduction

Topical and transdermal delivery of nonsteroidal anti-inflammatory drugs (NSAIDs) represents an important therapeutic strategy for the management of acute and chronic musculoskeletal pain, particularly in osteoarthritis, tendinopathies, and localized inflammatory conditions. Compared with oral administration, dermal application avoids first-pass hepatic metabolism, reduces systemic exposure, and thereby lowers the risk of gastrointestinal, cardiovascular, and renal adverse effects associated with prolonged systemic NSAID use (Pradal et al., 2019; European Medicines Agency, 2015). An additional advantage is ease of administration, which may improve patient adherence in the treatment of chronic pain conditions (Derry et al., 2016). Nevertheless, the therapeutic performance of topical products depends not only on the active pharmaceutical ingredient but also, to a large extent, on the dosage form, vehicle, and excipients, which govern drug release from the formulation, partitioning into the *stratum corneum*, and subsequent diffusion across the skin barrier (Benson, 2011).

*In vitro* permeation testing (IVPT) plays a central role in the early development of dermal formulations, formulation optimization, and comparison of the biopharmaceutical properties of different topical products. Such approaches provide controlled experimental conditions, good reproducibility, and reduced costs compared with *in vivo* studies (Lane, 2024). *Ex vivo* human skin is widely considered the benchmark barrier for IVPT and is explicitly recognized as an acceptable membrane in the Organization for Economic Co-operation and Development (OECD) Test Guideline 428 (OECD, 2004). However, the routine use of human skin is limited by ethical considerations, difficulties in sample procurement, restricted availability, and the need for appropriate tissue storage and validation. Owing to these limitations, there is a clear need for alternative models suitable for routine laboratory use (Neupane et al., 2020). In this context, routine laboratory use refers to models that can be used repeatedly in a standard formulation or analytical laboratory without primary tissue procurement, tissue banking, or case-by-case ethical approval, and that are readily available, inexpensive, and reproducible for regular deployment.

Two principal categories of surrogate permeation barriers have therefore been developed: synthetic membranes and cell-based models. Among synthetic membranes, Strat-M® is widely used as a multilayer non-biological system designed to mimic key transport characteristics of human skin, particularly the *stratum corneum* and viable epidermis, through a combination of controlled porosity, diffusion properties, and lipophilic/hydrophilic layers (Arce et al., 2020). Its major advantages include low inter-sample variability, standardized production, and convenient availability. Cell-based systems, such as epidermal constructs prepared from the immortalized human keratinocyte cell line (HaCaT), represent a biologically more complex alternative. HaCaT cells can be cultured at the air–liquid interface and express markers of terminal keratinocyte differentiation (Boukamp et al., 1988), which has motivated their use as an accessible epidermal model. Such systems may better reflect selected biological aspects of the epidermal environment, although they are generally more demanding in terms of preparation, characterization, and standardization (Neupane et al., 2020; Kichou et al., 2023). HaCaT-based epidermal equivalents may express terminal differentiation markers while forming only a modest and irregular stratum corneum, which limits their suitability as quantitative permeation barriers (Mini et al., 2021). Unlike commercially available human epidermis reconstruction kits, HaCaT-based constructs are inexpensive, readily available, and derived from a single immortalized keratinocyte line, making them attractive experimental systems; however, their use as quantitative permeation barriers requires construct-specific structural and functional characterization.

Most published studies evaluate individual models in isolation or compare different drugs, vehicles, or experimental conditions, which complicates the direct interpretation and transferability of findings across models (Ng et al., 2010; Finnin et al., 2011; Chedik et al., 2024). Consequently, limited guidance is available regarding the most appropriate application stage for each model and the extent to which data obtained across different barrier systems are comparable. This issue is particularly relevant for topical diclofenac, a widely used topical NSAID and a well-characterized, ionizable model permeant, as differences in composition, dosage form, salt form, and rheological properties have been shown to produce substantially different permeation profiles; therefore, drug concentration alone is insufficient to predict dermal absorption (Abd et al., 2016; OECD, 2025a). Comparative IVPT studies have evaluated Strat-M® against excised human skin and commercial models of reconstructed human epidermis (Kichou et al., 2023; Salminen et al., 2023). In contrast, published studies of HaCaT-based epidermal constructs have focused primarily on epidermal reconstruction, differentiation, and assessment of irritation or corrosion rather than on comparative IVPT of marketed topical drug products (Mini et al., 2021). To our knowledge, no previous study has directly compared Strat-M® and a HaCaT-based epidermal construct using marketed diclofenac gels under identical, internally controlled IVPT conditions. The specific contribution of the present study is therefore the direct comparison of the apparent permeation profiles and the capacity of the two barrier systems to discriminate between the same formulations. An epidermal model is appropriate for the present question because the principal diffusional resistance of the skin to most permeants lies in the stratum corneum and viable epidermis, and this study concerns this primary physical barrier. Full-thickness skin equivalents have their own place in such work: given an adequate physical barrier, they permit investigation of drug retention within the skin and distribution into the dermis, the site of action for locally acting topical NSAIDs. They address a different research objective from the one pursued here.

To facilitate a better understanding of the differences between Strat-M®, HaCaT-based epidermal equivalent, and two commonly used alternative models in permeation studies, EpiDerm™ (reconstructed human epidermis based on primary keratinocytes) and native human epidermis/skin as a reference model, their key characteristics are summarized in Table 1.

**TABLE 1:** Comparative characterization of the two barrier models used in this study (Strat-M^®^ and a HaCaT-based epidermal equivalent) against a reconstructed human epidermis model (EpiDerm™) and native human epidermis, compiled from published characterization studies and manufacturer documentation (Lane, 2024; OECD, 2004; Neupane et al., 2020; Kichou et al., 2023; Mini et al., 2021; OECD, 2025a; Haq et al., 2018; Kovács et al., 2021; Magnano et al., 2022; Boelsma et al., 1999; Jahn et al., 2024; Mattek, 2026; Ponec et al., 2000; Salminen et al., 2023; Schäfer Korting et al., 2008; Anjani et al., 2024; Kocsis et al., 2022; Sandby-Møller et al., 2003; Lundborg et al., 2018; Smits et al., 2017; Ya Xian et al., 1999; Holbrook and Odland, 1974; Kim et al., 2023; Ponec et al., 2002; Moran et al., 2021; Akdeniz et al., 2018; Kaur et al., 2018; Simon et al., 2016; Ribeiro et al., 2026; Netzlaff et al., 2007; OECD, 2025b; Marques et al., 2026).

Parameter	Strat-M®	HaCaT construct	EpiDerm™ (RHE, primary keratinocytes)	Native human epidermis/skin (reference)
Model identity and architecture				
1. Model type, origin, cell source, and presence of viable cells	Synthetic multilayer membrane; polyethersulfone layers over a polyolefin support, impregnated with a proprietary lipid blend. No cells, no metabolic or repair capacity.^a^ ^,^ ^c^ (Haq et al., 2018; Kovács et al., 2021; Magnano et al., 2022)	Epidermal equivalent reconstructed from the spontaneously immortalized, aneuploid human keratinocyte line HaCaT. Viable cells present.^b^ (Boelsma et al., 1999; Jahn et al., 2024)	Commercial reconstructed human epidermis from primary human keratinocytes cultured at the air–liquid interface; viable cells present. EpiDerm-200-X was used in the FDA parallel evaluation.^a^ ^,^ ^b^ (Mattek, 2026; Ponec et al., 2000; Salminen et al., 2023; Schäfer Korting et al., 2008)	Excised human skin or heat-separated human epidermis; viability depends on preparation and storage. Reference barrier.^b^ ^,^ ^c^ (Lane, 2024; Salminen et al., 2023)
2. Total thickness and rate-limiting barrier layer (µm)	Total thickness ≈300–325 µm. Resistance arises from the tight uppermost polyethersulfone layer and lipid impregnation; the diffusion path is a tortuous synthetic matrix rather than ordered intercellular lipid lamellae.^a^ ^,^ ^b^ (Kovács et al., 2021; Magnano et al., 2022; Anjani et al., 2024)	Not reported for the present construct. An independent HaCaT-based epidermal equivalent showed a stratum corneum described as modest (Mini et al., 2021); a separate comparison found HaCaT-derived epidermis thinner than that from primary keratinocytes and devoid of stratum granulosum and corneum, so no defined rate-limiting corneal layer.^b^ ^,^ ^†^ (Mini et al., 2021; Jahn et al., 2024)	Total tissue thickness 70–130 µm (mean 111.0 µm), with at least four viable cell layers (EPI-200 lot histology); supplier specification 8–12 cell layers plus stratum corneum. The rate-limiting barrier is the stratum corneum.^a^ ^,^ ^b^ (Mattek, 2026)	Stratum corneum 18.3 ± 4.9 µm (dorsal forearm), 11.0 ± 2.2 µm (shoulder), 14.9 ± 3.4 µm (buttock); viable epidermis 56.6 ± 11.5, 70.3 ± 13.6, and 81.5 ± 15.7 µm, respectively (n = 71 donors). The rate-limiting barrier is the stratum corneum.^b^ (Magnano et al., 2022; Sandby Møller et al., 2003; Lundborg et al., 2018)
3. Stratum corneum: presence, continuity, and corneocyte layer number	No true stratum corneum. A tight surface layer and lipid coating simulate stratum corneum function; corneocytes are absent. Corneocyte layer number not applicable.^a^ ^,^ ^b^ (Haq et al., 2018; Anjani et al., 2024)	Discrepant between laboratories. Two independent reports describe HaCaT-derived epidermis as devoid of stratum granulosum and corneum, or a stratum corneum as lacking (Jahn et al., 2024; Smits et al., 2017); a third reports a stratum corneum that is present but modest and irregular (Mini et al., 2021). Corneocyte layer number not reported. The discrepancy indicates strong protocol dependence rather than a single characteristic value.^b^ ^,^ ^†^ (Mini et al., 2021; Jahn et al., 2024; Smits et al., 2017)	Characteristic stratum corneum present; extracellular lipid lamellae observed, but not regular throughout the full stratum corneum. Corneocyte layer number not reported.^a^ ^,^ ^b^ (Mattek, 2026; Ponec et al., 2000; Salminen et al., 2023)	True stratum corneum present. The number of corneocyte layers is strongly site-dependent, ranging from approximately 50 layers in palmoplantar skin to fewer than 10 in facial and genital skin; corneocytes are approximately 30 µm in diameter and 0.5–0.8 µm thick.^b^ (Lundborg et al., 2018; Ya Xian et al., 1999; Holbrook and Odland, 1974)
Barrier composition and function				
4. Stratum corneum lipid composition and lamellar organization (class level)	Proprietary synthetic lipid blend applied in ratios intended to resemble human stratum corneum; pores are lipid-impermeabilized. Organization is synthetic rather than biological lamellae; ceramide, free fatty acid, and cholesterol profiles are not reported.^a^ ^,^ ^c^ (Haq et al., 2018; Magnano et al., 2022)	Impaired barrier-lipid synthesis reported for HaCaT constructs: free fatty acids absent, ceramides very low and incomplete, and abnormal stratum corneum lipid organization. No quantitative lipid profiling of HaCaT epidermal equivalents was identified. Not measured in the present construct.^c^ ^,^ ^†^ (Boelsma et al., 1999; Kim et al., 2023)	All major epidermal lipid classes are present. Relative to native epidermis, polar ceramides 5 and 6 are lower, ceramide 7 is absent, and free fatty acids are very low; the long periodicity phase is present, but the short periodicity phase is missing.^b^ (Ponec et al., 2000; Ponec et al., 2002)	Ceramides, cholesterol, and free fatty acids are arranged in organized stacked bilayers/lamellae; the composition most closely matching this arrangement best reproduces permeability behavior. (Lundborg et al., 2018)
5. Epidermal differentiation status and organization	Not applicable — no viable cells and no epidermal differentiation programme.^a^ (Haq et al., 2018)	Abnormal and incomplete terminal differentiation. K1/K10 expression irregular, K6/K16 present in all layers; transcription of cornified-envelope proteins (filaggrin, loricrin, involucrin) abnormal relative to primary keratinocytes; karyotype aneuploid. One HaCaT-based equivalent expressed CK10, CK14, involucrin, and filaggrin, but only at a modest level in the stratum corneum. Differentiated HaCaT cells can upregulate KRT1, KRT10, and IVL under some conditions, but three-dimensional barrier maturation remains weak.^b^ ^,^ ^†^ (Mini et al., 2021; Boelsma et al., 1999; Jahn et al., 2024; Smits et al., 2017; Kim et al., 2023)	Completely stratified epithelium closely resembling normal human epidermis, although differentiation-marker expression is aberrant for K6, involucrin, and transglutaminase.^b^ (Ponec et al., 2002)	Normal terminal differentiation generates the stratum corneum from keratinocytes; native structure is the benchmark for organization and barrier maturation.^b^ (Lundborg et al., 2018)
6. Barrier integrity metrics: TEER, TEWL	Not applicable — TEER and TEWL are viable-tissue integrity readouts. Not reported for Strat-M®, which is used as a permeation membrane rather than a barrier-readout system.^b^ ^,^ ^d^ (Kovács et al., 2021)	HaCaT monolayers did not form a robust barrier: no increase in TEER followed calcium-induced differentiation, in contrast to primary human foreskin keratinocytes and N/TERT2G cells, both of which increased. In three-dimensional comparisons, TEER was lower than for better-performing immortalized lines. Absolute TEER values not reported.^b^ ^,^ ^†^ (Jahn et al., 2024; Moran et al., 2021)	TEWL was elevated relative to excised human skin for EpiDerm-200-X; absolute values were reported graphically only. Selected in earlier validation work as the RHE with the best barrier properties among those tested.^b^ (Salminen et al., 2023; Schäfer Korting et al., 2008)	Pooled TEWL reference values for healthy adults range from 2.3 g m^-2^ h^-1^ (95% CI 1.9–2.7; breast) to 44.0 g m^-2^ h^-1^ (95% CI 39.8–48.2; axilla) across 86 skin areas in 212 studies. IVPT integrity acceptance thresholds are laboratory- and guideline-dependent (e.g., <10 or <15 g m^-2^ h^-1^) rather than universal.^b^ ^,^ ^c^ (Lane, 2024; Salminen et al., 2023; Akdeniz et al., 2018)
7. Reported permeation behavior, probe compounds, and correlation with human skin	Good correlation with human cadaver skin for nicotine formulations; closer to human skin than EpiSkin® for resorcinol flux; diclofenac formulation trends correlated with human skin equivalent; amphotericin B R2 = 0.99 and f2 = 85; rivastigmine profile similar to pig skin (R2 = 0.920) with IVIVC R2 = 0.991, though lower than human skin in later work. In the FDA parallel evaluation, the lowest permeation was observed for all three probes. Permeability coefficients not reported.^b^ (Kichou et al., 2023; Haq et al., 2018; Kovács et al., 2021; Salminen et al., 2023; Kaur et al., 2018; Simon et al., 2016; Ribeiro et al., 2026)	Lucifer yellow was not retained and passed completely through HaCaT-derived epidermis, whereas other immortalized lines excluded the dye comparably to primary keratinocytes, indicating poor barrier function. No permeability coefficient or drug permeation data identified.^b^ ^,^ ^†^ (Jahn et al., 2024)	6 h cumulative permeation of a finite dose (6 nmol cm^-2^) was highest in EpiDerm-200-X for caffeine and testosterone (EpiDerm > EHS > Strat-M®), whereas salicylic acid permeated most in EHS (EHS > EpiDerm > Strat-M®). RHE permeation generally exceeds that of human epidermis, but the substance ranking reflects this. Permeability coefficients not reported.^b^ (Salminen et al., 2023; Schäfer Korting et al., 2008)	Reference standard. 6 h cumulative permeation in EHS: caffeine 0.97 ± 0.22, testosterone 0.33 ± 0.13, and salicylic acid 0.18 ± 0.10 nmol cm-2 (log Kow −0.07, 3.32, and 2.26, respectively). Human skin equivalent discriminated caffeine from testosterone, whereas Episkin did not; for the rivastigmine IVIVC, human skin yielded the highest correlation coefficient among the membranes tested.^b^ (Salminen et al., 2023; Ribeiro et al., 2026; Netzlaff et al., 2007)
8. Known sources of variability	Low batch-to-batch variation, straightforward storage and handling, reproducible performance. Compound- and formulation-dependent deviations from human skin nonetheless persist.^a^ ^,^ ^b^ (Kovács et al., 2021; Anjani et al., 2024; Kaur et al., 2018)	Donor variation is avoided because the line is immortalized, but architecture and differentiation depend strongly on the protocol —calcium concentration, culture duration, time at the air–liquid interface, and passage number. Reported stratum corneum status ranges from absent to modest and irregular between laboratories, so no single representative value can be given.^b^ ^,^ ^†^ (Mini et al., 2021; Boelsma et al., 1999; Jahn et al., 2024; Smits et al., 2017)	Low intra-batch variation, but moderate to considerable inter-batch variation in tissue architecture and marker expression; reproducibility not clearly superior to excised skin.^b^ (Schäfer Korting et al., 2008; Ponec et al., 2002)	Varies with donor, anatomical site, collection, preparation, handling, and storage; intra- and inter-lot variability of up to 37% and 50%, respectively, has been reported.^c^ (Lane, 2024; Magnano et al., 2022)
Application to *in vitro* permeation testing				
9. Demonstrated formulation-discrimination capability	Demonstrated for nicotine/CPE formulations, diclofenac hydrogels versus creams, rivastigmine transdermal patches across membranes, and dissolving microarray patches.^b^ (Haq et al., 2018; Kovács et al., 2021; Anjani et al., 2024; Ribeiro et al., 2026)	Not demonstrated. The available literature addresses cell biology and barrier weakness rather than comparative formulation discrimination.^b^ ^,^ ^d^ ^,^ ^†^ (Boelsma et al., 1999; Jahn et al., 2024)	Supports permeation ranking across substances and formulations; selected for finite-dose work as the RHE with the best barrier properties among those tested.^b^ (Salminen et al., 2023; Schäfer Korting et al., 2008)	The comparator for formulation discrimination and for regulatory IVPT and bioequivalence work.^c^ (Lane, 2024; Salminen et al., 2023)
10. Reproducibility, availability, and regulatory/ethical status	Commercial, non-animal, readily available, long shelf life, and reproducible; useful for reducing the use of human and animal tissue. No regulatory validation as a skin-permeation surrogate.^a^ ^,^ ^b^ (Neupane et al., 2020; Kovács et al., 2021; Kaur et al., 2018; Simon et al., 2016)	Readily cultured, immortalized line with no donor constraints and low cost. Not validated for any regulatory purpose and not established as a permeability surrogate.^c^ ^,^ ^d^ (Boelsma et al., 1999; Jahn et al., 2024)	Commercially available. RHE models are regulatory-validated for skin irritation (OECD TG 439) and skin corrosion (OECD TG 431). Not validated for dermal absorption: OECD TG 428 specifies the use of excised human or animal skin. Reproducibility is not markedly better than that of excised skin.^a^ ^,^ ^c^ (OECD, 2004; OECD, 2025a; Schäfer Korting et al., 2008; OECD, 2025b)	Limited supply, high cost, ethical and logistical constraints, and an integrity-control burden. Remains the accepted reference standard under OECD TG 428 and IVPT guidance.^c^ (Lane, 2024; OECD, 2004; Neupane et al., 2020)
11. Principal limitation and appropriate scope of use	Absence of viable biology and a tortuous synthetic diffusion path rather than ordered stratum corneum lipid lamellae, so absolute prediction is imperfect. Role: standardized comparative screening and formulation ranking under defined conditions, and reduction of biological membrane use, prior to confirmatory testing.^b^ (Haq et al., 2018; Anjani et al., 2024; Simon et al., 2016)	Incomplete terminal differentiation and absent or, at best, modest and irregular stratum corneum formation, providing no adequate physical barrier for quantitative permeation. Role: standardized comparative ranking where a viable, stratified barrier is required; not a replacement for human skin where absolute permeability matters.^b^ ^,^ ^†^ (Mini et al., 2021; Mattek, 2026; Smits et al., 2017)	Higher permeability than native epidermis and non-native lipid organization; validated for irritation and corrosion but not for absorption. Role: standardized comparative ranking where a viable, stratified barrier is required; not a replacement for human skin where absolute permeability matters.^b^ (Ponec et al., 2000; Salminen et al., 2023; Schäfer Korting et al., 2008)	Limitation: supply, cost, donor and preparation variability, and integrity-control burden. Role: final reference standard for confirmatory IVPT and bioequivalence-relevant testing.^c^ (Lane, 2024; Salminen et al., 2023; Marques et al., 2026)

Provenance of data

^a^ Manufacturer documentation.

^b^ Experimentally measured in a peer-reviewed study.

^c^ Reported in the literature without primary measurement.

^d^ Not reported.

^†^ Reported for HaCaT-derived epidermal equivalents in the cited studies; not measured in the present construct. The construct used here was not characterized for barrier function, lipid composition, or stratum corneum architecture, and no property in this column should be read as a measured property of it.

“Not reported” indicates that the parameter was not reported in the sources consulted; “not applicable” indicates that the parameter has no meaningful value for that model. No value has been inferred from a related model.

Numerical values are given as reported ranges or as mean ± SD, with sample size consistently reported. Units are stated in every case.

Permeability coefficients (Kp, cm h^-1^) were not reported for any of the four models in the sources consulted; row7 therefore summarises reported permeation behavior, probe compounds, and correlation statistics rather than harmonized coefficients.

Where published findings for HaCaT constructs conflict (rows 2, 3, and 8), both are presented rather than reconciled, since the divergence reflects genuine protocol dependence of stratum corneum formation in this class of model.

Abbreviations: CPE, chemical penetration enhancer; EHS, excised human skin; IVIVC, *in vitro–in vivo* correlation; IVPT, *in vitro* permeation testing; Kow, octanol–water partition coefficient; Kp, permeability coefficient; RHE, reconstructed human epidermis; TEER, transepithelial/transepidermal electrical resistance; TEWL, transepidermal water loss.

The aim of this pilot comparative study was to perform a direct, internally controlled comparison of the synthetic Strat-M® membrane and a HaCaT-based epidermal construct using Voltaren® and Olfen®, two marketed, nominally dose-equivalent diclofenac gels, under otherwise identical IVPT conditions. We evaluated whether the two barrier models yielded consistent apparent permeation profiles and estimates of steady-state flux (Jss), lag time (*t*lag), and permeability coefficient (Kp), and whether they led to the same comparative interpretation of the formulations. The overall experimental workflow is summarised in Figure 1.

**FIGURE 1:**
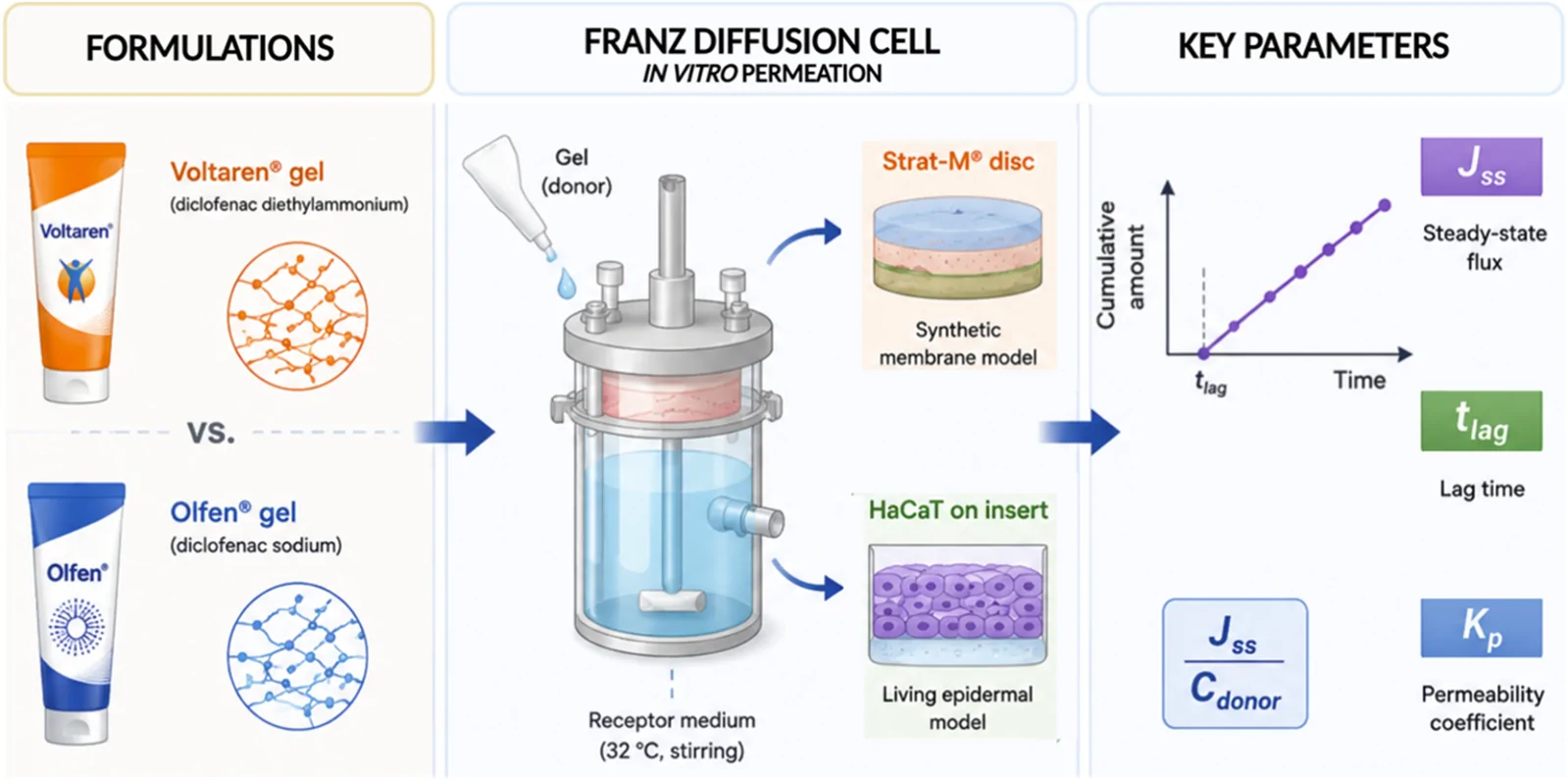
Experimental workflow of the comparative IVPT study. Two commercial diclofenac gels, Voltaren® and Olfen®, were evaluated using Franz diffusion cells and two structurally distinct barrier models: Strat-M® and the HaCaT construct. The main permeation outputs included cumulative permeated amount, Jss, *t*lag, and Kp. The study was designed to assess whether the selection of a barrier model influences the comparative interpretation of formulation performance. Created in BioRender.com.

## 2 Materials and methods

### 2.1 Materials

Two commercially available topical diclofenac formulations were used in this study; Voltaren Emulgel® 11.6 mg/g gel, containing diclofenac diethylammonium, and Olfen® 10 mg/g gel, containing diclofenac sodium. Voltaren Emulgel® was manufactured by GlaxoSmithKline Dungarvan Limited, Knockbrack, Dungarvan, Co. Waterford, Ireland; batch no. 8C8D, expiry May 2026. Olfen® gel was manufactured by Pliva Ljubljana d.o.o., Ameriška ulica 2, 1,000 Ljubljana, Slovenia; batch no. C58579A, expiry May 2028. Both products were purchased from a community pharmacy and stored according to the manufacturers’ specifications until use. Both formulations were within their declared shelf life at the time of the experiments. When expressed as diclofenac sodium equivalents on the basis of molecular weight (Mw) conversion (diclofenac diethylammonium Mw 369.29 g/mol; diclofenac sodium Mw 318.13 g/mol), the diclofenac diethylammonium content of 11.6 mg/g in Voltaren Emulgel® corresponds to approximately 10.0 mg/g of diclofenac sodium, making the two formulations nominally equivalent in terms of active substance concentration.

Diclofenac sodium salt (cat. no. D6899, LOT BCBS5961V, CAS 15307-79-6, Mw 318.13 g/mol, purity ≥98% (TLC); Sigma-Aldrich, St. Louis, MO, United States) was used as the reference standard for calibration. The standard was stored protected from light at room temperature.

Strat-M® synthetic membranes (25 mm discs, trial pack; cat. no. SKBM025TP, LOT 0000488378, expiry September 2025; Merck Millipore Ltd., Tullagreen, Carrigtwohill, Co. Cork, Ireland) were stored at room temperature in their original sealed pouch until use. All membranes were used within the manufacturer’s declared shelf life.

HaCaT keratinocytes, kindly provided by Prof. Dr. Elsa Fabbretti, University of Primorska, Slovenia, were used to prepare the HaCaT-based construct. Cell culture reagents included Advanced Dulbecco’s Modified Eagle Medium/Ham’s F-12 (Advanced DMEM/F-12; Gibco™, Thermo Fisher Scientific, Waltham, MA, United States), fetal bovine serum (FBS; Gibco™, Thermo Fisher Scientific), L-glutamine (ReagentPlus, cat. no. G3126, CAS 56-85-9; Sigma-Aldrich), penicillin G sodium salt (BioReagent, CAS 69-57-8; Sigma-Aldrich), and streptomycin sulfate salt (CAS 3810-74-0; Sigma-Aldrich). Advanced DMEM/F-12 supplemented with 5% (w/w) FBS, 2 mmol/L L-glutamine, 100 U/mL penicillin G, and 1 mg/mL streptomycin is hereafter referred to as complete Advanced DMEM/F-12. Ultrapure water (18.2 MΩ·cm at 25 °C; Fluka, Germany) was used to prepare antibiotic and L-glutamine stock solutions. Nunc™ tissue culture flasks (Thermo Fisher Scientific, Bremen, Germany) were used for routine cell maintenance. Corning® 6-well cell culture inserts (cat. no. 3450; Corning Inc., Corning, NY, United States) with PET membranes, 0.4 µm pore size, and 4.67 cm^2^ growth area were used for preparation of the HaCaT-based construct, and 24-well cell culture inserts (cat. no. 3470; Corning Inc., Corning, NY, United States) were used for morphological and immunocytochemical characterization. Trypsin, phosphate-buffered saline (PBS), bovine serum albumin (BSA), 4% formaldehyde, Triton X-100, and Tween 20 were obtained from Sigma-Aldrich.

Reagents used for morphological and immunocytochemical characterization were as follows. F-actin was visualized with CytoPainter Phalloidin-iFluor 555 Reagent (cat. no. ab176756; Abcam, Cambridge, UK). Primary antibodies were directed against filaggrin (cat. no. ab81468; Abcam) and loricrin (cat. no. ab198994; Abcam). Secondary antibodies were goat anti-rabbit IgG (cat. no. ab150077; Abcam) and goat anti-mouse IgG (cat. no. ab150113; Abcam). Nuclei were counterstained using DAPI-containing mounting medium (cat. no. ab104139; Abcam, Cambridge, UK).

The commercial reconstructed human epidermis MatTek EpiDerm™ (cat. no. EPI-200-EACH) and the corresponding assay medium (cat. no. EPI-100-ASY) were obtained from MatTek (Bratislava, Slovakia) and served exclusively as a qualitative reference model for epidermal morphology and differentiation marker expression; this model was not included in the permeation experiments.

The qualitative compositions of the two marketed gels, compiled from the manufacturers’ Summaries of Product Characteristics and package inserts, are summarized in Table 2 (Voltaren, 2026; Olfen, 2026). Although both products deliver a nominally equivalent dose of 10 mg/g expressed as diclofenac sodium equivalents, they differ in salt form, dosage form, gelling polymer, and several excipients with recognized permeation-enhancing potential, while sharing purified water and isopropyl alcohol as common vehicle components. Because the SmPCs disclose excipients qualitatively but not quantitatively, the relative concentrations of individual components could not be compared, and no independent analytical determination of excipient identity or content was performed. This pair of marketed products, therefore, provides a practical test case for assessing whether the Strat-M® and HaCaT barrier models detect formulation-driven differences in apparent diclofenac permeation.

**TABLE 2:** Composition of the two commercial diclofenac gels evaluated in the present study, compiled from manufacturers’ SmPCs and packaging inserts (Voltaren, 2026, Olfen, 2026).

Component	Voltaren ® Emulgel 11.6 mg/g gel	Olfen ® 10 mg/g gel
Active substance/salt/conc.	Diclofenac diethylammonium/11.6 mg/g (equivalent to 10 mg/g diclofenac sodium)	Diclofenac sodium/10 mg/g
Vehicle/co-solvents	Purified water, isopropyl alcohol, propylene glycol (E1520)	Purified water, isopropyl alcohol
Gelling agent	Carbomer	Hydroxyethyl cellulose, hydroxypropyl cellulose
Formulation type	Emulgel, consisting of a biphasic oil-in-water system incorporated into a gel matrix	Simple hydrogel formulation
Permeation enhancers (recognised)	Propylene glycol, isopropyl alcohol, cocoyl caprylocaprate, macrogol cetostearyl ether	Diisopropyl adipate, isopropyl alcohol
Other notable excipients	Diethylamine, liquid paraffin, perfume cream 45 (contains allergens: benzyl alcohol, benzyl benzoate, etc.)	Sodium metabisulfite (E223), lactic acid

### 2.2 Preparation of the Strat-M ® barrier

Each Strat-M® membrane was visually inspected for defects before mounting. Strat-M® is a synthetic multilayer membrane approximately 300 µm thick, consisting of two polyethersulfone layers of higher diffusional resistance supported by a more open polyolefin layer, with a proprietary blend of synthetic lipids impregnated throughout the porous structure (Arce et al., 2020). Membranes were clamped directly between the glass flanges of the Franz diffusion cell without additional sealant, in accordance with the manufacturer’s instructions, with the shiny, skin-mimicking polyethersulfone side facing the donor compartment and the polyolefin side facing the receptor compartment. The absence of air bubbles beneath the membrane was verified visually. No pre-hydration or equilibration step was applied before formulation application, as the manufacturer specifies that the membrane requires no such pretreatment. All Strat-M® membranes originated from the same lot.

Strat-M® membranes were therefore mounted in the dry, as-supplied state, whereas HaCaT barriers were mounted in the hydrated, as-cultured state (Section 2.3). This difference in initial hydration was inherent to the two model systems and was deliberately retained, as each barrier was used under the condition for which it is intended.

### 2.3 Culture and barrier formation of the HaCaT-based epidermal construct

HaCaT cells were routinely maintained in complete Advanced DMEM/F-12 at 37 °C in a humidified atmosphere containing 5% CO_2_ in Nunc™ tissue culture flasks until confluence.

For seeding onto inserts, confluent HaCaT cells were trypsinized, centrifuged at 300 *g* for 5 min, and resuspended in complete Advanced DMEM/F-12.

Cells were seeded onto PET membrane inserts in 6-well plates at a density of 1.5 × 10^6^ cells per insert. The apical compartment received 1.5 mL of cell suspension, and the basolateral compartment received 2.5 mL of complete Advanced DMEM/F-12. During the submerged phase, cells were cultured for 6 days at 37 °C in a humidified atmosphere with 5% CO_2_ until complete surface coverage was achieved. Medium was replaced every 2–3 days in both compartments.

After 6 days, the air–liquid interface (ALI) was established to promote keratinocyte differentiation and maturation of the HaCaT construct. The apical medium was aspirated, and the apical surface was left exposed to air. During the ALI phase, medium was supplied only to the basolateral compartment, with replacement every 2–3 days. The ALI phase was maintained for 14–15 days at 37 °C in a humidified atmosphere with 5% CO_2_.

After a total culture period of 20–21 days (6 days submerged +14–15 days ALI), the insert membrane bearing the ALI-cultured HaCaT construct was carefully excised from the insert housing with a scalpel and transferred to a standard 6-well plate (without inserts) containing 1 mL of complete Advanced DMEM/F-12 to prevent desiccation of the cell layer before mounting in the Franz diffusion cell. The excised membrane (4.67 cm^2^ growth area, corresponding to a diameter of approximately 24 mm) fully covered the 20 mm orifice of the Franz cell (3.14 cm^2^ diffusion area) and was mounted with the apical cell surface facing the donor compartment. Each HaCaT-bearing membrane was visually inspected after excision and before mounting to exclude gross macroscopic damage. However, no functional barrier integrity measurements, such as TEER or tracer permeability, were performed after excision or mounting in the Franz diffusion cell.

Each formulation was tested in triplicate on the HaCaT barrier model. The three HaCaT barriers used for each formulation were prepared within the same culture experiment and mounted in separate Franz diffusion cells; therefore, they were treated as technical replicates. Independent biological culture batches were not included in this pilot study.

### 2.4 Morphological characterization and epidermal differentiation of the HaCaT construct

HaCaT cells were cultured on inserts in 24 – well plates and maintained under air–liquid interface (ALI) conditions for the duration of the experiment. Following completion of the ALI culture phase, representative HaCaT–bearing inserts were fixed with 4% formaldehyde for 10 min and washed three times for 5 min each with PBS. Cell morphology, cytoskeletal organization, and confluence were assessed by phalloidin and DAPI staining, while epidermal differentiation was assessed by immunocytochemical staining for filaggrin and loricrin.

For F-actin staining, fixed samples were incubated with phalloidin diluted 1:1,000 in PBS containing 1% BSA for 90 min at room temperature in the dark. Samples were then washed three times for 5 min with PBS and mounted in DAPI-containing mounting medium, and imaged after 5 min.

For immunocytochemical staining, fixed samples were permeabilized with 0.1% Triton X-100 in PBS for 10 min and washed three times for 5 min with PBS. Non-specific binding was blocked with PBS containing 1% BSA and 0.1% Tween 20 for 30 min at room temperature. Samples were then incubated overnight at 4 °C with primary antibodies against filaggrin and loricrin, each diluted 1:200 in PBS containing 1% BSA and 0.1% Tween-20. After three washes with PBS, samples were incubated with the corresponding secondary antibody diluted 1:500 in PBS containing 1% BSA for 1 h at room temperature in the dark, washed three times for 5 min with PBS, and mounted in DAPI-containing mounting medium. Fluorescence microscopy was performed using an EVOS FL microscope (Thermo Fisher Scientific) at ×10 magnification.

The MatTek EpiDerm™ reference model was processed in parallel. On receipt, inserts were transferred to a 6-well plate containing 900 µL of assay medium and equilibrated overnight at 37 °C in a humidified atmosphere with 5% CO_2_. Inserts were subsequently transferred to a 24-well plate, rinsed with PBS, fixed with 4% formaldehyde for 10 min, washed three times for 5 min each with PBS, and stained with phalloidin and immunocytochemical protocols as described above. Representative images are presented in the Results section (Figure 2).

**FIGURE 2:**
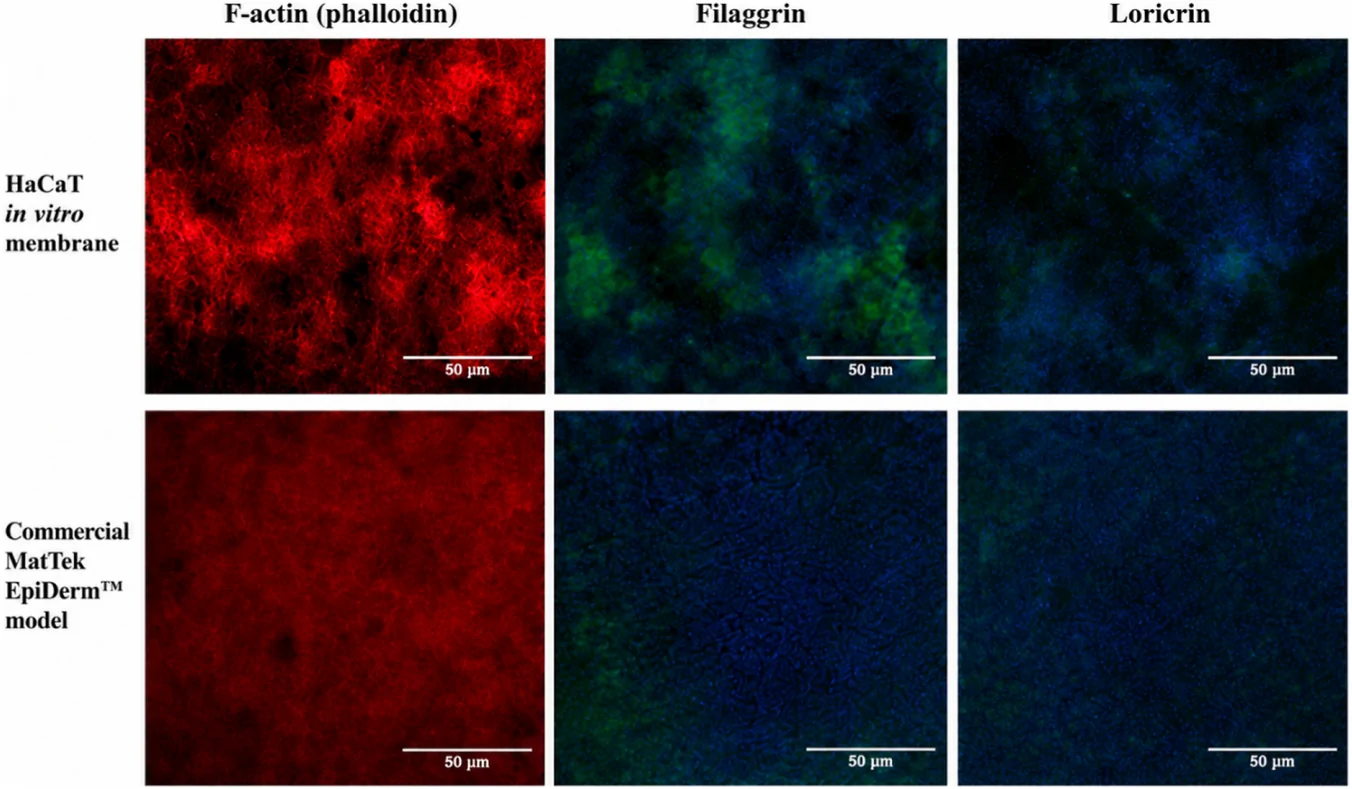
Morphological characterization and epidermal differentiation of the HaCaT construct (top row) and the commercial reconstructed human epidermis MatTek EpiDerm™ (bottom row). The left column shows F-actin (phalloidin, red) with DAPI nuclear counterstaining (blue); the middle column shows filaggrin (green) with DAPI; and the right column shows loricrin (green) with DAPI. Images were acquired using an EVOS FL fluorescence microscope at ×10 magnification. Scale bars represent 50 μm.

All characterization was performed on *en face* preparations; cross–sectional imaging and measurement of layer thickness were not undertaken.

### 2.5 *In vitro* permeation study (IVPT)

Permeation experiments were performed using an automated Franz diffusion cell system (Logan System 912-6; Logan Instruments Corp., Somerset, NJ, United States) equipped with vertical glass diffusion cells with a diffusion area of 3.14 cm^2^ and a receptor compartment volume of 15 mL. Complete Advanced DMEM/F-12 was used as the receptor medium in all permeation experiments. The same receptor medium was used for both barrier models to maintain comparability across experimental conditions and to preserve HaCaT viability during the permeation study. Before the experiment, the receptor medium was pre-warmed to 32 °C, filled into the receptor compartment, thermostated at 32 °C ± 0.5 °C, and continuously stirred throughout the study. A temperature of 32 °C was selected to approximate the physiological surface temperature of human skin.

Each formulation was weighed onto a silicone carrier with a surface area of 1.7 cm^2^ and transferred onto the barrier surface. After application, the gel spread beyond the initial carrier boundaries, covering the entire available diffusion area of the Franz cell. Each formulation was tested in triplicate on both barrier models, yielding a total of 12 permeation experiments. The mean applied masses were 582.0 ± 14.1 mg for Olfen® and 571.9 ± 8.2 mg for Voltaren Emulgel® on Strat-M®, and 531.0 ± 3.5 mg for Olfen® and 529.0 ± 3.6 mg for Voltaren Emulgel® on HaCaT. These masses correspond to approximately 168–185 mg of gel per cm^2^, equivalent to approximately 1.7–1.9 mg of diclofenac (expressed as diclofenac sodium equivalents) per cm^2^. The applied dose greatly exceeded the conventional finite-dose range; the experiments were therefore conducted under infinite-dose conditions, in which the donor concentration is assumed to remain approximately constant. Over the 24 h experimental period, the cumulative amount of diclofenac recovered in the receptor compartment corresponded to at most approximately 19% of the applied dose. A mass balance was not performed, and this assumption was therefore not verified experimentally; Kp values are consequently reported as apparent values (Section 4.6). The donor compartment was sealed with Parafilm® M to limit solvent evaporation and maintain constant experimental conditions.

At predefined time points of 0.5, 1, 2, 3, 4, 5, 6, and 24 h, aliquots of 1.0 mL were automatically withdrawn from the receptor compartment by the integrated autosampler. Each withdrawn sample was immediately replaced with the same volume of fresh, pre-warmed complete Advanced DMEM/F-12 to maintain a constant receptor volume.

Sink conditions were evaluated retrospectively. The highest diclofenac concentration measured in the receptor compartment at any time point corresponded to approximately 73 µg/mL, which represents less than 1% of the reported aqueous solubility of diclofenac sodium (approximately 30 mM, equivalent to ≈9.5 mg/mL, at 25 °C) (Fini et al., 2012). Sink conditions were therefore assumed to be maintained throughout the experiment, although the saturation solubility of diclofenac sodium in the specific receptor medium used (complete Advanced DMEM/F-12 supplemented with 5% FBS) was not experimentally determined.

### 2.6 Quantification by UV–Vis spectrophotometry

Diclofenac concentrations in receptor samples were determined by UV–Vis spectrophotometry (Cary 60 UV–Vis; Agilent Technologies, Santa Clara, CA, United States) at 289 nm using quartz cuvettes with a 10 mm optical path length. The wavelength of 289 nm was selected to reduce spectral overlap with the absorption band of serum proteins in the FBS-containing receptor medium (λmax ≈280 nm). Complete Advanced DMEM/F-12 was used as the blank to compensate for the medium matrix’s background absorbance. All samples were measured in triplicate.

Calibration standards were prepared by dissolving the diclofenac sodium reference standard in complete Advanced DMEM/F-12 at seven concentrations: 1, 4, 10, 22, 28, 34, and 40 µg/mL. Each level was measured in triplicate. The calibration was linear over the working range of 4–40 µg/mL, corresponding to 0.004–0.040 mg/mL, with the regression equation A = 27.338 × C + 0.0696 (R^2^ = 0.991), where A is the absorbance at 289 nm, and C is the diclofenac sodium concentration expressed in mg/mL. The 1 µg/mL calibration level was excluded from the working range because the back-calculated recovery fell outside acceptable limits. Intra-day precision, expressed as relative standard deviation (RSD), was below 3.4% at all concentration levels within the working range.

The limit of quantification (LOQ) was experimentally determined as the lowest calibration level that met the accuracy and precision criteria, in accordance with ICH Q2(R2). On this basis, the LOQ was set at 4 µg/mL because all calibration levels from 4 µg/mL upward met these criteria, whereas the 1 µg/mL level did not. For comparison, calculation based on the residual standard deviation of the calibration regression and its slope (LOD = 3.3 × σ/S and LOQ = 10 × σ/S) yielded estimated values of 5.2 and 15.9 µg/mL for the LOD and LOQ, respectively. Because the experimentally established lower limit was directly evaluated for accuracy and precision, 4 µg/mL was used as the operational LOQ for the present study.

Receptor concentrations below the experimentally established LOQ occurred only at the earliest sampling points. When back-calculation yielded values at or below the calibration intercept, the corresponding concentrations were set to zero for cumulative permeation calculations; no sampling time points were removed from the dataset. Because numerical treatment of early below-LOQ observations may influence the regression intercept and, hence, the estimated *t*lag, a sensitivity analysis was additionally performed by substituting 0, LOQ/2, LOQ/√2, and LOQ (Supplementary Table S1). This analysis was intended to assess the sensitivity of the estimated *t*lag to the handling of below-LOQ data, rather than to identify a preferred substitution method.

The method was matrix-matched, as calibration standards were prepared in the same receptor medium as the samples, thereby inherently accounting for background absorbance from the serum-containing medium.

All cumulative permeated amounts are reported as diclofenac sodium equivalents. Since diclofenac (pKa ≈4.0) is more than 99.9% ionized at the receptor medium pH of 7.4, the UV-absorbing species in the receptor compartment is the diclofenac anion, irrespective of the salt form present in the donor formulation.

### 2.7 Calculation of permeation parameters

Sample concentrations were calculated from the calibration curve according to the following equation:

$$C=\frac{A-0.0696}{27.338}$$

where C is the diclofenac sodium concentration in mg/mL and A is the absorbance at 289 nm.

The cumulative mass of permeated diclofenac (Q) was calculated from the measured concentrations, accounting for the 15 mL receptor volume and correcting for diclofenac removed in the 1.0 mL aliquots withdrawn and replaced with fresh medium at each sampling time point. This cumulative mass was then normalized to the available active diffusion area of the Franz diffusion cell (A = 3.14 cm^2^), which was completely covered by the formulation following application. Furthermore, the normalized cumulative proportion of permeated diclofenac (%) was calculated using the exact initial weight and concentration of the applied formulation. Standard deviations (SD) and coefficients of variation (CV) were determined for all of the aforementioned permeation parameters: concentration (C), cumulative mass (Q), normalized cumulative mass (Q/A), and the normalized cumulative portion of permeated diclofenac (%).

Here, Q_1_ and Q_2_ denote cumulative permeated masses, A is the diffusion area (3.14 cm^2^), and time is expressed in hours when reporting Jss in μg cm^−2^ h^−1^:

$$J_{ss}=\frac{\left(Q_{2}-Q_{1}\right)}{\left[A\left(t_{2}-t_{1}\right)\right]}$$

*T* lag was estimated by extrapolating the linear portion of the cumulative permeation curve to the time axis. The permeability coefficient (Kp, cm h^-1^) was calculated from the Jss and donor concentration according to the following equation:

$$K_{p}=\frac{J_{ss}}{C_{d}}$$

where Cd was approximated as 10 mg/mL, corresponding to 10,000 µg/mL, based on the declared diclofenac sodium-equivalent concentration in both formulations. Mean values, SD, and coefficients of variation (CV) were calculated from the three replicates for each formulation and barrier model.

For each individual diffusion cell, the coefficient of determination ( $R^{2}$ ) was obtained by fitting a linear regression to the cumulative permeated amount per unit area as a function of time, using all data points within the predefined linear interval. The same cell-specific interval was used to calculate Jss and to estimate *t*lag by extrapolation to the time axis. The selected regression windows and corresponding $R^{2}$ values for all individual diffusion cells are provided in Supplementary Table S2.

### 2.8 Statistical analysis

All statistical evaluations were performed using JASP (version 0.19.1; JASP Team, University of Amsterdam, Netherlands). Comparisons were performed using independent-samples Welch’s *t*-tests, selected to account for possible unequal variances between groups, with statistical significance set at *p* < 0.05. Because of the pilot design and the small number of replicates (*n* = 3 per group), comparisons were interpreted as exploratory and were supplemented with effect-size estimates and confidence intervals. Two-sided 95% confidence intervals (CIs) were calculated for both mean differences and Hedges’ *g*. Results are reported as the mean difference (95% CI), *p*-value, and Hedges’ *g* (95% CI), with differences calculated as the first-listed group minus the second-listed group (Supplementary Table S3).

An approximate sensitivity analysis was performed using the observed within-group variability in the HaCaT groups, with a pooled within-group standard deviation of Jss of approximately 8.10 µg cm^-2^ h^-1^. With *n* = 3 per formulation and a two-sided test at α = 0.05, the smallest between-formulation difference in Jss that this design could have detected with 80% probability was approximately 25 µg cm^-2^ h^-1^, substantially larger than the difference observed on the HaCaT construct. This analysis is approximate, since a standard deviation estimated from three replicates is itself imprecise; it indicates the order of magnitude of the difference the study could resolve and is not a *post hoc* power calculation. The non-significant HaCaT comparison should therefore be interpreted as inconclusive rather than as evidence of equivalent permeation.

## 3 Results

### 3.1 En-face characterization of the HaCaT construct

Before use in permeation experiments, representative HaCaT constructs were evaluated by *en face* morphological and immunocytochemical imaging and qualitatively compared with the commercial reconstructed human epidermis, MatTek EpiDerm™ (Figure 2). Phalloidin and DAPI staining of the HaCaT construct revealed a continuous, dense cell layer with a well-organized actin cytoskeleton, in which DAPI-stained nuclei were distributed across the entire analyzed surface without prominent cell-free areas, consistent with a high degree of confluence (Figure 2). A comparably continuous cell layer was observed in the MatTek EpiDerm™ model (Figure 2).

Positive staining for filaggrin and loricrin was observed in both models (Figures 2). In the HaCaT construct, the signals for both differentiation markers were clearly present but exhibited spatial heterogeneity, whereas the MatTek EpiDerm™ model displayed a more uniform distribution across the analyzed surface. Filaggrin is expressed during late keratinocyte differentiation, whereas loricrin is a marker of terminal epidermal differentiation.

The *en face* fluorescence images showed a continuous HaCaT cell-covered surface with detectable expression of the late- and terminal-differentiation markers filaggrin and loricrin. However, because no cross-sectional imaging, thickness measurement, or quantitative barrier-function assessment was performed, these findings demonstrate surface continuity and expression of differentiation markers but do not establish multilayer architecture, stratum corneum formation, or functional barrier competence.

### 3.2 Diclofenac permeation profiles

The cumulative permeation curves (Figures 3a,b) showed a characteristic profile for all conditions: an initial lag phase followed by pseudo-steady-state permeation. On the Strat-M® membrane, the profiles of the two formulations diverged, whereas on the HaCaT construct, they overlapped throughout the sampling period. Additionally, the lag phase was consistently longer on the Strat-M® membrane than on HaCaT.

**FIGURE 3:**
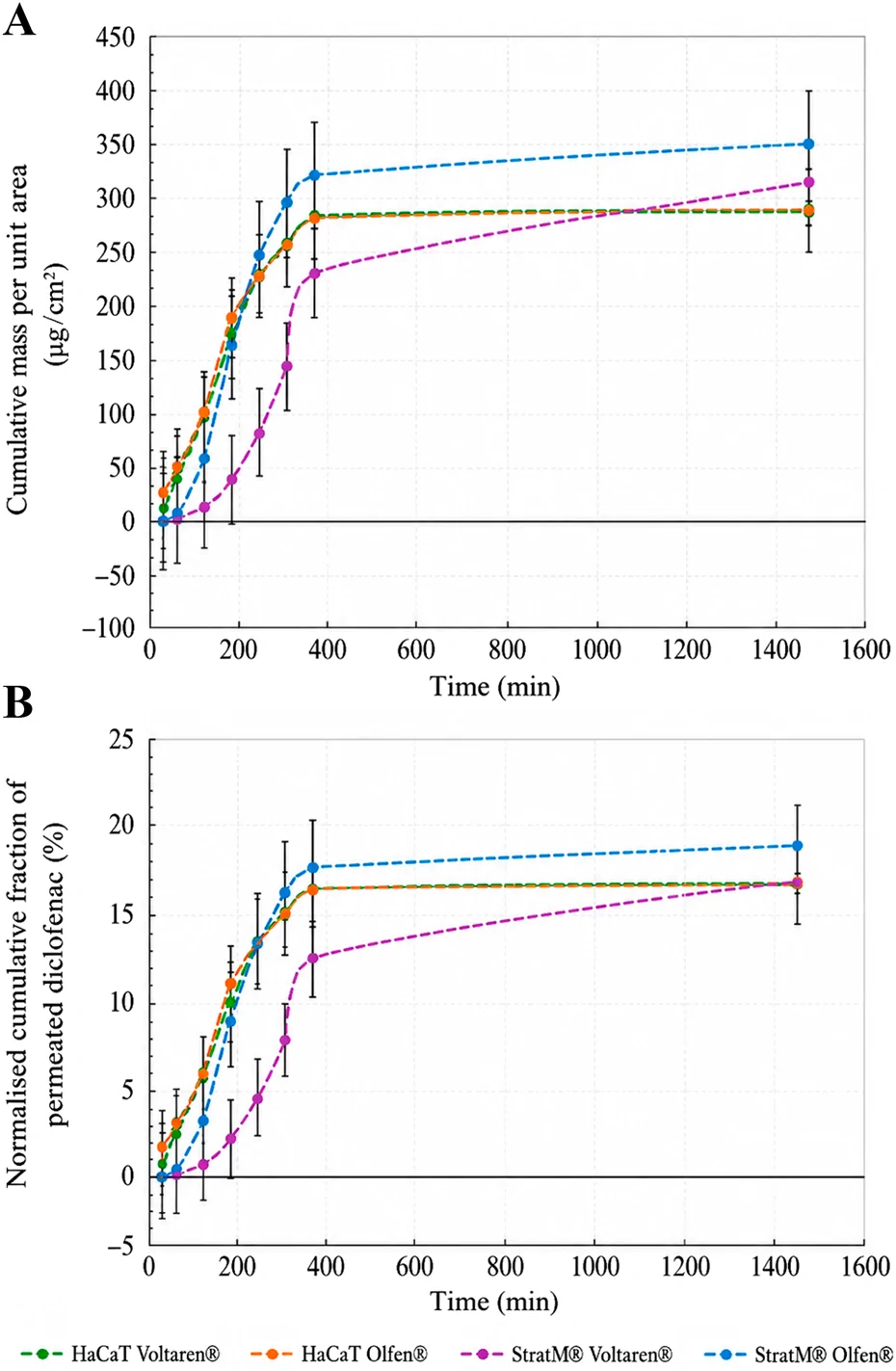
Permeation of diclofenac from Voltaren® and Olfen® gels across HaCaT and Strat-M® barrier models (mean ± SD, n = 3). **(A)** cumulative mass of permeated diclofenac per unit area over time; **(B)** normalized cumulative fraction of permeated diclofenac over time.

### 3.3 Permeation parameters

The permeation parameters derived from these profiles are summarized in Table 3. The only statistically significant difference between the two formulations was observed for Jss on the Strat-M® membrane, where Olfen® exceeded Voltaren® by approximately 1.8-fold; no corresponding difference was detected on the HaCaT construct. Conversely, *t*lag differed significantly between the two barrier models for both formulations, whereas Jss did not.

**TABLE 3:** Permeation parameters for Voltaren^®^ and Olfen^®^ gels across the HaCaT and Strat-M^®^ barrier models. Data are expressed as mean ± SD (n = 3 technical replicates per group). Comparisons were performed using independent samples Welch’s t-tests, with statistical significance set at p < 0.05.

Barrier model	Formulation	Jss (µgcm^-2^h^-1^)	*t*lag (h)	Kp × 10^–3^ (cmh^-1^)
HaCaT Strat-M®	Voltaren® Olfen® Voltaren® Olfen®	66.35 ± 8.08 62.20 ± 8.11 48.03 ± 10.38* 85.30 ± 14.90 *	0.49 ± 0.31^#^ 0.24 ± 0.08^#^ 2.23 ± 0.69^#^ 1.21 ± 0.39^#^	6.63 ± 0.81 6.22 ± 0.81 4.80 ± 1.04 8.53 ± 1.49

* Statistically significant difference (p < 0.05) between formulations within the same barrier model. On Strat-M®, Jss was approximately 1.8-fold higher for Olfen® than for Voltaren® (mean difference 37.3 µgcm-2h-1, 95% CI, 6.72 to 67.81; p = 0.029), whereas the two formulations did not differ significantly in Jss on HaCaT (p = 0.565).

^#^ Statistically significant difference (p < 0.05) between barrier models for the same formulation. *T*lag was significantly longer on Strat-M® than on HaCaT for both Voltaren® (p = 0.033) and Olfen® (p = 0.046). In contrast, between-model differences in Jss did not reach significance for either formulation (Voltaren®: p = 0.077; Olfen®: p = 0.097), and *t*lag did not differ significantly between the two formulations within either barrier model.

Kp was calculated as Jss/Cd using the same nominal donor concentration (10 mg/mL) for both formulations and both models. Kp is therefore directly proportional to Jss, and no separate statistical testing was performed on Kp.

### 3.4 Effect of formulation within each model

The effect of the formulation was inconsistent between the two barrier models. In the HaCaT construct, the two gels yielded similar Jss (66.35 ± 8.08 vs. 62.20 ± 8.11 µg cm^-2^ h^-1^), and the difference was not statistically significant (mean difference, Olfen® − Voltaren®, −4.14 µg cm^-2^ h^-1^, 95% CI −22.50 to 14.22; p = 0.565; Hedges’ g −0.41). On the Strat-M® membrane, by contrast, Olfen® produced a significantly higher Jss than Voltaren® (85.30 ± 14.90 vs. 48.03 ± 10.38 µg cm^-2^ h^-1^; mean difference, Olfen® − Voltaren®, 37.26 µg cm^-2^ h^-1^, 95% CI 6.72 to 67.81; p = 0.029; Hedges’ g 2.32 ), corresponding to an approximately 1.8-fold difference (Table 3; Supplementary Table S3). *T*lag did not differ significantly between the two formulations within either barrier model.

The ordering of the mean Jss values also differed between the models: Voltaren® gave the higher mean on HaCaT, whereas Olfen® gave the significantly higher value on Strat-M®. Because the difference in HaCaT was not statistically significant, this reversal is reported solely as a difference in the ordering of mean values between barriers and does not support any ranking of the two formulations.

### 3.5 Effect of barrier model within each formulation

*T*lag was the parameter most sensitive to barrier model: for both formulations, replacing HaCaT with Strat-M® increased *t*lag significantly, from 0.49 ± 0.31 h to 2.23 ± 0.69 h for Voltaren® (an approximately 4.5-fold increase; p = 0.033) and from 0.24 ± 0.08 h to 1.21 ± 0.39 h for Olfen® (an approximately 5-fold increase; p = 0.046), corresponding to a shift from roughly 15–30 min to roughly 70–135 min.

By contrast, between-model differences in Jss did not reach statistical significance for either formulation (Voltaren®: p = 0.077; Olfen®: p = 0.097), and the direction of the effect was not consistent: on switching from HaCaT to Strat-M®, mean Jss decreased for Voltaren® (66.35–48.03 µg cm^-2^ h^-1^) but increased for Olfen® (62.20–85.30 µg cm^-2^ h^-1^).

Across both formulations, barrier model selection therefore affected *t*lag consistently and substantially, whereas its effect on Jss was smaller, non-significant, and dependent on the formulation tested.

## 4 Discussion

### 4.1 Model-dependent discrimination between two commercial diclofenac gels

Strat-M® yielded a statistically significant between-formulation difference in Jss, whereas no statistically significant difference was detected with the HaCaT construct. This is consistent with literature emphasizing the discriminatory power of Strat-M® for evaluating topical formulations; Milanowski et al. reported that Strat-M® provides reproducible *in vitro* permeation parameters for topical NSAIDs and captures formulation-driven differences in drug release (Milanowski et al., 2021). In the present study, although the two commercial gels were nominally comparable in their declared active-substance content, their Jss differed significantly on the synthetic membrane (85.30 ± 14.90 vs. 48.03 ± 10.38 µg cm^-2^ h^-1^; p = 0.029).

Across the Strat-M® membrane, Olfen® exhibited an approximately 1.8-fold higher Jss of diclofenac than Voltaren®. This is consistent with the notion that synthetic membranes containing defined lipophilic and hydrophilic polymeric domains respond sensitively to vehicle composition and salt form. In contrast, the two formulations produced closely similar mean Jss values on the HaCaT construct (66.35 ± 8.08 vs. 62.20 ± 8.11 µg cm^-2^ h^-1^; p = 0.565), but the confidence interval was wide (−22.50 to 14.22 µg cm^-2^ h^-1^), and the comparison was inconclusive rather than evidence of equivalent permeation. No independent cellular or biological endpoint was measured in the HaCaT arm. Because Kp was calculated as Jss using the same nominal donor concentration throughout, it follows Jss exactly and provides no independent information.

### 4.2 Possible formulation-related basis for the higher Jss of Olfen® than Voltaren® on Strat-M®

The excipient compositions were available only qualitatively, from the SmPCs and package inserts, and no independent analytical characterization of the formulations was performed; the following interpretation is therefore hypothesis-generating rather than mechanistic.

Although the two products are nominally comparable in diclofenac content when expressed as diclofenac sodium equivalents, they differ in dosage form, gelling polymer, and several excipients. Voltaren® is a carbomer-based emulgel containing diclofenac diethylammonium, propylene glycol, liquid paraffin, cocoyl caprylocaprate, and macrogol cetostearyl ether, whereas Olfen® is a cellulose-based hydrogel containing diclofenac sodium and diisopropyl adipate. Isopropyl alcohol is present in both products and therefore cannot, in itself, account for the observed difference, because the SmPCs disclose excipients qualitatively but not quantitatively, and differences in the amounts of individual components could not be assessed (Voltaren, 2026; Olfen, 2026). These formulation differences may influence diclofenac solubility in the donor phase, its thermodynamic activity, release from the gel matrix, and partitioning into the membrane. Previous work has shown that Strat-M® responds sensitively to the thermodynamic state of the drug in the vehicle, such that modest changes in excipient composition can shift flux profiles substantially (Moran et al., 2021; Manian et al., 2022); however, the contribution of individual formulation variables was not measured here.

The importance of vehicle composition is supported by Pradal et al. (Pradal et al., 2019), although the direction of their findings differed from that observed here. In their study using *ex vivo* human skin, a diclofenac diethylamine 1.16% emulsion permeated more extensively at 24 h than a diclofenac sodium 5% gel, despite the latter containing a higher concentration of the active substance. In the present study, by contrast, the diclofenac sodium product (Olfen®) produced the highest Jss on Strat-M®. This apparent discrepancy is informative: it indicates that diclofenac permeation cannot be predicted from salt form or drug concentration alone but depends on the complete formulation system and on the properties of the barrier used. Because Strat-M® and human skin differ structurally, the membrane result should not be interpreted as a direct prediction of permeation through human skin (Pradal et al., 2019; Moran et al., 2021).

Published studies indicate that salt form may influence saturation solubility, dissolution, and membrane transport, although the resulting permeation behavior depends on the complete formulation (Neupane et al., 2020; Manian et al., 2022). Similarly, the structure of the gelling matrix may affect drug release from the vehicle (Pradal et al., 2019). Although propylene glycol is generally recognized as a permeation enhancer, its contribution in Voltaren® cannot be inferred from the present results.

Formulation pH, rheology, thermodynamic activity, quantitative excipient composition, and actual drug content were not determined, and no mass balance was performed (Section 4.6); the higher Jss of Olfen® on Strat-M® can therefore not be attributed to any individual component or specific mechanism. The present findings support only the broader conclusion that nominally comparable diclofenac concentrations do not necessarily produce comparable apparent permeation profiles, and that the outcome depends on the complete formulation-barrier system.

### 4.3 Interpretation of the HaCaT permeation results

To place the present comparison in the context of established barrier characteristics, Table 1 summarizes the structural, compositional, and functional properties of the two barriers used here, along with a validated reconstructed human epidermis (EpiDerm™) and native human epidermis, compiled from published characterization studies. The comparison provides a structural and functional context for interpreting why different permeation behaviors may arise across the two model types. Strat-M® substitutes a tight polyethersulfone layer impregnated with a synthetic lipid blend for the stratum corneum and reproduces human-skin permeation across a broad physicochemical range with low lot-to-lot variability. A HaCaT construct, by contrast, has been reported to form at best a modest and irregular stratum corneum, and in other reports, no stratum corneum at all (Table 1). Even a validated primary keratinocyte model such as EpiDerm™, although fully stratified with a functional lipid-containing stratum corneum, is more permeable than native skin and exhibits an altered ceramide profile. Our two barriers, therefore, sit at opposite ends of a gradient; from an acellular but barrier-competent synthetic membrane, through an immature HaCaT layer, to a fully stratified but supra-permeable reconstructed epidermis. The observed differences are consistent with the substantially different structural and barrier properties reported for these model types. However, because the functional integrity and cross-sectional architecture of the present HaCaT construct were not measured, neither its lack of a statistically significant between-formulation difference nor its shorter *t*lag can be attributed to a specific structural or transport mechanism. Recent comparison of a full-thickness 3D *in vitro* model with *ex vivo* human skin likewise showed that model-specific permeability may preserve relative permeation trends while differing in absolute permeability, underscoring the need to define and validate the intended use of each model (Marques et al., 2026).

The magnitude of the difference between the formulation means also differed between the barriers. On HaCaT, the two gels differed by approximately 7% (66.35 ± 8.08 vs. 62.20 ± 8.11 µg cm^-2^ h^-1^), whereas on Strat-M®, the means differed by approximately 1.8-fold. With three replicates per group and a wide confidence interval, the non-significant HaCaT result is inconclusive and does not establish that the formulation effect was small. Within-group coefficients of variation for Jss were 12%–13% on HaCaT and 18%–22% on Strat-M®.

Within-group variability in *t*lag was similar across three of the four experimental conditions (CV approximately 31%–32%) but was markedly higher for Voltaren® on the HaCaT construct (CV approximately 62%). Potential sources of variability in cell-based constructs include differences in seeding, culture conditions, differentiation, hydration, and handling; however, the individual contributions of these factors were not assessed in the present study. Schäfer-Korting et al., in a validation study of reconstructed epidermal models for skin absorption, also reported intra-laboratory variability in biological barrier systems (Schäfer Korting et al., 2008). In the present dataset, the elevated variability in *t*lag was therefore confined primarily to the Voltaren®/HaCaT condition rather than being a consistent feature of the HaCaT construct across both formulations. Because independent culture batches and post-mounting integrity measurements were not included, the source of this higher variability cannot be identified.

Two constraints bound this interpretation. Expression of filaggrin and loricrin confirms selected aspects of keratinocyte differentiation but does not demonstrate a physiologically competent stratum corneum. The spatial heterogeneity of the staining indicates that differentiation was not uniform across the analyzed surface, which may have been accompanied by spatially variable transport resistance. In addition, functional integrity was not quantified before or after mounting: cross-sectional architecture and thickness, transepithelial electrical resistance or tracer permeability, lipid and tight junction organization, and inter-batch reproducibility were not assessed. The present data, therefore, do not validate this construct as a quantitative IVPT barrier, and neither its lack of formulation discrimination nor its shorter lag time can be assigned to a specific mechanism.

### 4.4 Interpretation of the longer *t*lag on Strat-M®

Across both formulations, the diclofenac *t*lag was consistently longer with Strat-M® than with the HaCaT construct. For Voltaren® Emulgel, *t*lag increased from 0.49 ± 0.31 h on HaCaT to 2.23 ± 0.69 h on Strat-M® (p = 0.033), and for Olfen® from 0.24 ± 0.08 h to 1.21 ± 0.39 h (p = 0.046). Thus, *t*lag was approximately 4.5-fold (Voltaren®) and 5.0-fold (Olfen®) longer on the synthetic membrane and represented the permeation parameter most sensitive to barrier model selection in the present dataset (Table 3). By contrast, within either barrier model, the two formulations did not differ significantly in *t*lag.

The longer *t*lag on Strat-M® is consistent with its architecture and dimensions. Strat-M® is approximately 300 µm thick and consists of two polyethersulfone layers of higher diffusional resistance, which face the donor compartment, supported by a more open polyolefin layer, with synthetic lipids impregnated throughout the porous structure (Arce et al., 2020). This manufactured structure provides a defined diffusional pathway. By contrast, no cross-sectional thickness measurement was performed for the HaCaT construct, so the effective thickness of the HaCaT/PET system cannot be compared directly with Strat-M® or used to quantitatively explain the observed difference in tlag. In the classical Fickian description of diffusion through a homogeneous membrane, *t*lag is proportional to membrane thickness squared and inversely proportional to the diffusion coefficient (*t*lag = h^2^/6D) (Crank, 1979); however, application of this relationship to the present cross-model comparison remains qualitative because the HaCaT/PET system was neither homogeneous nor structurally quantified. The internal architecture of the two barrier systems is compared schematically in Figure 4.

**FIGURE 4:**
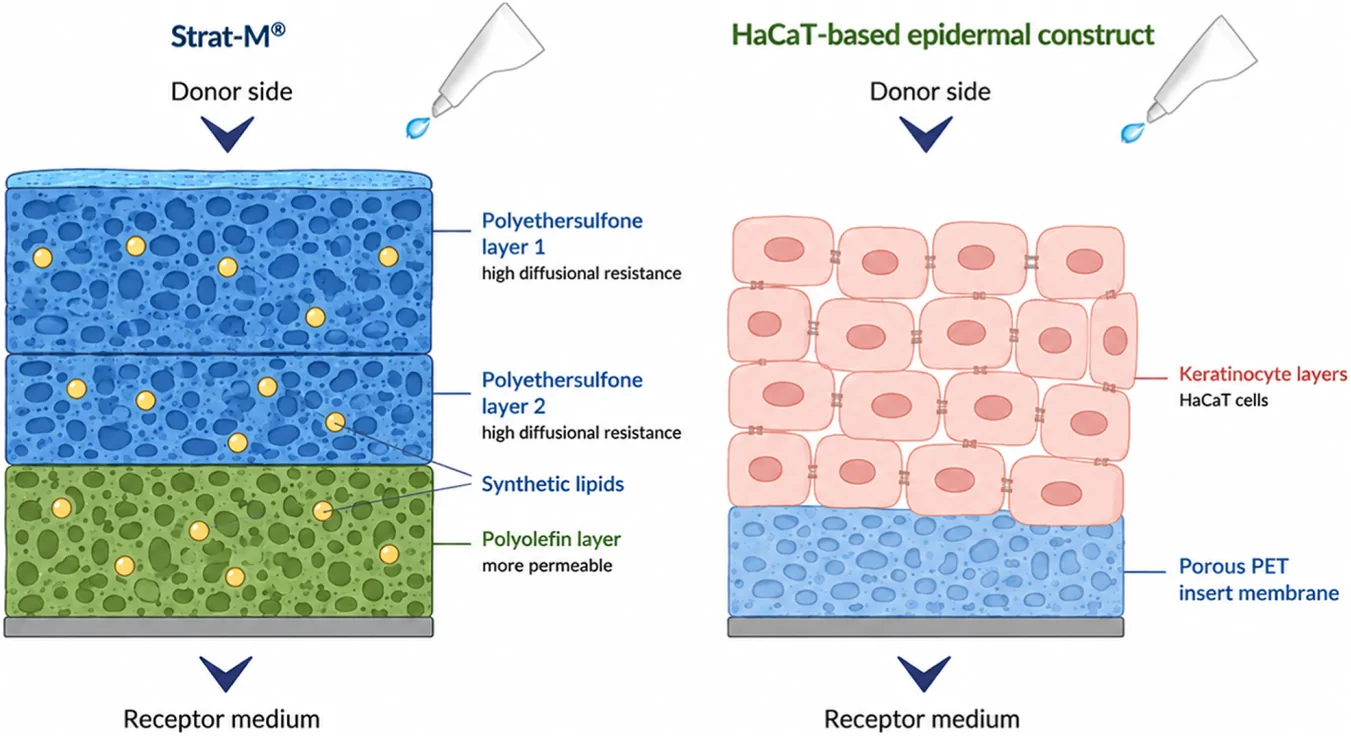
Schematic representation of the two barrier systems as mounted in the Franz diffusion cell. In both panels, the formulation is applied to the donor side (top), and the receptor medium lies below. Left: the Strat-M® synthetic membrane, comprising two polyethersulfone layers of high diffusional resistance over a more permeable polyolefin support, with synthetic lipids distributed throughout the porous structure; this depiction follows the manufacturer’s description of the membrane. Right: the HaCaT construct, comprising keratinocytes cultured at the air–liquid interface on a porous PET insert membrane. The right-hand panel is a generic depiction of this class of model and not a representation of the construct used here: no cross-sectional imaging was performed, multilayer architecture was not confirmed, and neither the number nor the arrangement of the cell layers shown was measured in this study. No stratum corneum is depicted, and none was demonstrated. The drawing is not to scale and is intended solely to contrast the architectural principles of the two model types. Created in BioRender.com.

Initial membrane hydration may also have contributed to the longer *t*lag. Strat-M® membranes were stored dry and used without pre-hydration, in accordance with the manufacturer’s instructions. Before steady-state transport could be established, the porous polymeric and lipid-impregnated domains may have required progressive wetting, equilibration, and saturation. During this early phase, components of the donor vehicle and receptor medium may have contributed to membrane hydration rather than to the immediate transport of diclofenac. Comparisons of dry and pre-equilibrated synthetic membranes indicate that the absence of initial hydration extends the interval required for a permeant to establish a stable concentration gradient across the membrane (Moran et al., 2021; Farmacia, 2019). In contrast, the HaCaT construct was maintained in a fully hydrated culture environment before mounting in the Franz diffusion cell, which may have facilitated faster initial partitioning and diffusion.

The shorter *t*lag observed with HaCaT may reflect differences in hydration and architecture between the two systems; however, handling-related microdamage and incomplete barrier maturation cannot be excluded. First, because barrier integrity was not tested at any stage, handling-related microdamage introduced during excision, transfer, and clamping may have contributed (Section 4.6). Second, because the degree of stratification and the presence of a stratum corneum were not established, an immature barrier remains a plausible contributor rather than one that can be ruled out. Positive filaggrin and loricrin staining establishes that differentiation markers were expressed; it does not establish that a competent stratum corneum was formed. Reconstructed epidermal barriers have been reported to be more hydrated than synthetic polymeric membranes (Hagvall et al., 2023).

Vehicle composition may have contributed to the observed *t*lag differences, although its contribution cannot be quantified from the present data. Within either barrier model, the two formulations did not differ significantly in *t*lag, whereas the barrier model effect was significant for both formulations. Taken together, these observations suggest that differences in barrier architecture, effective diffusional path length, hydration state, and potentially functional integrity may all contribute to the observed differences in *t*lag. Their individual contributions cannot be distinguished within the present experimental design (Manian et al., 2022; Farmacia, 2019).

An analytical limitation should also be considered. Receptor concentrations at the earliest sampling points on Strat-M® were below the LOQ and were set to zero for cumulative permeation calculations. These observations occurred at or before the start of the corresponding pseudo-steady-state regression intervals and therefore did not affect Jss or Kp. Because their numerical treatment may influence the regression intercept and hence *t*lag, a sensitivity analysis using substitutions of 0, LOQ/2, LOQ/√2, and LOQ was performed. The model-dependent difference in *t*lag remained evident across all tested substitutions (Supplementary Table S1).

In summary, *t*lag was consistently longer with Strat-M® than with the HaCaT construct. Differences in architecture, effective diffusional path length, and initial hydration may have contributed to this result; however, because the structure and functional integrity of the HaCaT construct were not quantified, the mechanism responsible for the observed difference cannot be established from the present data.

### 4.5 Practical implications for fit-for-purpose barrier-model selection

The present study has practical implications for the design and interpretation of comparative IVPT studies. Under otherwise identical experimental conditions, Strat-M® detected a statistically significant difference in steady-state flux between Olfen® and Voltaren®, whereas the HaCaT construct did not demonstrate a statistically significant difference between formulations; lag time was also consistently shorter with the HaCaT construct (Table 3). For the specific objective of discriminating between the two formulations based on apparent flux, the HaCaT construct as implemented here did not provide additional discriminatory information beyond that obtained with Strat-M®. These findings reinforce that comparative permeation outcomes must be interpreted in relation to the barrier model used, as synthetic membranes, reconstructed epidermal models, and excised skin differ in their structure, composition, and transport properties (Lane, 2024; Neupane et al., 2020; Kichou et al., 2023; Chedik et al., 2024).

For early formulation screening, Strat-M® offers a practical and standardized platform for controlled comparisons. Its ready-to-use format and manufactured multilayer structure facilitate reproducible testing without the biological variability and tissue-sourcing requirements associated with excised skin (Neupane et al., 2020; Arce et al., 2020; Kichou et al., 2023; Magnano et al., 2022; Mattek, 2026). In the present study, Strat-M® distinguished Olfen® from Voltaren® in terms of apparent steady-state flux, supporting its utility in detecting formulation-dependent differences under defined experimental conditions. However, comparative results obtained with Strat-M® remain specific to that membrane and should not be interpreted as direct estimates of permeation through human skin, because the relationship between Strat-M® and human skin permeability varies with the permeant and formulation tested (Neupane et al., 2020; Kichou et al., 2023; Mattek, 2026). Where IVPT data are intended to support biorelevant, equivalence, or regulatory conclusions, testing should follow an appropriately justified strategy that uses validated methodology and a qualified biological barrier, most commonly excised human skin (European Medicines Agency, 2015; Lane, 2024; OECD, 2004).

The HaCaT construct may serve a distinct role within a fit-for-purpose experimental framework. HaCaT cells are an established keratinocyte model, and HaCaT-based systems have been applied to directly measure cellular and tissue-related endpoints, including viability, irritation, and responses to topical exposure (Boukamp et al., 1988; Mini et al., 2021). Such applications should be distinguished from quantitative permeation testing, for which the functional barrier properties and reproducibility of the specific construct must be established. Published comparisons demonstrate that differentiation-marker expression alone does not necessarily predict functional barrier competence and that barrier assessment may require complementary structural and functional endpoints, such as cross-sectional morphology, tracer permeability, or electrical resistance (Mini et al., 2021; Abd et al., 2016; Jahn et al., 2024). The present findings therefore support selecting each model according to the scientific question and the evidence available for its intended use, rather than following a fixed sequence in which different barriers are assumed to provide equivalent or inherently complementary permeation information.

From a pharmaceutical-development perspective, this fit-for-purpose approach can improve both the interpretation of comparative data and the allocation of experimental resources. Standardized synthetic membranes can support initial formulation screening, cell-based systems can address directly measured keratinocyte-related endpoints, and validated reconstructed epidermis or qualified excised skin can be used when greater biological or regulatory relevance is required (European Medicines Agency, 2015; Lane, 2024; OECD, 2004; Neupane et al., 2020; Kichou et al., 2023; Chedik et al., 2024). This approach may reduce the risk of advancing or rejecting a formulation based solely on its behavior in a single surrogate barrier. Accordingly, the selected model, its intended role, and the limits of the conclusions that can be drawn from it should be defined prospectively and reported explicitly.

### 4.6 Strengths and limitations

This pilot study was designed as an internally controlled comparison in which the same active ingredient, the same two commercial gels, and the same Franz diffusion cell apparatus, receptor medium, temperature, and analytical method were applied across both barrier models. This design minimized methodological variability and isolated formulation type and barrier model as the principal variables. Both positive and negative outcomes are reported transparently, which is appropriate for a pilot comparison aimed at defining the applicability of each model.

As expected for a pilot investigation, the scope was deliberately focused, and the findings should be interpreted within this context. Two marketed formulations were tested in triplicate (twelve experiments in total). This design was sufficient to reveal the relatively large model-dependent differences observed in this study, but had limited power to detect smaller between-formulation effects. The three HaCaT constructs used for each formulation were generated within a single culture experiment and therefore represent technical replicates rather than independent biological replicates. Inter-batch reproducibility was not assessed in the present study. Reproducibility across independent culture batches should therefore be evaluated prospectively using predefined acceptance criteria, together with functional barrier qualification and cross-sectional characterization, before the construct is used for confirmatory comparative IVPT or formulation decision-making. Accordingly, the HaCaT results and associated statistical comparisons reflect within-batch variability only and do not capture potential biological variability between independent culture batches.

The HaCaT construct was characterized only by *en face* morphology and immunocytochemical detection of filaggrin and loricrin. Cross-sectional histology with thickness measurement was not performed, and additional functional assays (e.g., TEER, lipid profiling, tight junction markers) were not conducted. Cross-sectional structural characterization and functional barrier qualification are prerequisites for the use of the construct in confirmatory quantitative IVPT rather than optional extensions. Barrier integrity was not tested before or after mounting, so handling-related microdamage during excision, transfer, and clamping cannot be excluded as a contributor to the observed results. The HaCaT permeation data should therefore be interpreted as apparent transport through the construct under the experimental conditions used, rather than as transport through a functionally validated epidermal barrier. Future work should apply predefined functional-integrity acceptance criteria using TEER and/or a suitable tracer-permeability assay before and after mounting. In addition, benchmarking against excised human skin would be required to establish the biorelevance of either surrogate barrier. *Ex vivo* human-skin IVPT was not undertaken because it would require prior institutional ethics committee approval, a compliant tissue-supply or donor-consent pathway, and the associated documentation and agreements, which together constitute a separate prospective study. MatTek EpiDerm™ served exclusively as a qualitative morphological and differentiation-marker reference and was not included as an IVPT comparator. The study, therefore, cannot determine whether the apparent permeation profiles obtained with the HaCaT construct would resemble or differ from those of an established reconstructed human epidermis under comparable conditions; the qualitative *en face* comparison does not substitute for a direct permeation comparison.

No complete mass-balance assessment was performed. Only diclofenac measured in the receptor compartment was quantified; residual formulation in the donor compartment, drug retained within the Strat-M® membrane or HaCaT/PET construct, and drug associated with other diffusion-cell components were not recovered or analyzed. Total recovery and the distribution of diclofenac within the experimental system could therefore not be established. In particular, the present data cannot distinguish receptor-side permeation from retention within the HaCaT cell layer or the PET support, or from losses elsewhere in the system. The reported Kp values should accordingly be regarded as apparent values calculated under the infinite-dose assumption rather than as values supported by experimental confirmation of constant donor concentration or complete recovery.

Finally, several analytical and formulation-related limitations apply. The excipient composition of the two gels was obtained qualitatively from the SmPCs (Table 2) and was not determined analytically; quantitative excipient analysis, formulation characterization (pH, rheology, thermodynamic activity of diclofenac in each vehicle), and direct assay of drug content would be required to attribute the observed flux difference to specific formulation variables. Diclofenac was quantified by a matrix-matched UV–Vis method. A few early receptor concentrations fell below the LOQ and were set to zero for cumulative permeation calculations. Their treatment did not affect Jss or Kp, while sensitivity analysis confirmed that the model-dependent difference in *t*lag remained evident across alternative numerical treatments of the below-LOQ observations (Supplementary Table S1). Kp values were based on declared label content and a single membrane lot. Replacement of UV–Vis spectrophotometry with a separation-based chromatographic method (HPLC or UPLC) would reduce the potential for residual excipient interference in confirmatory work.

## 5 Conclusion

This pilot study provided a direct, internally controlled comparison of diclofenac permeation from Voltaren® and Olfen®, two marketed, nominally dose-equivalent topical gels, using Strat-M® and a HaCaT construct under harmonised IVPT conditions. Strat-M® detected a statistically significant difference in steady-state flux between the formulations, whereas the HaCaT construct did not show a statistically significant difference between formulations. Lag time was consistently shorter with the HaCaT construct. Thus, the two barriers generated different apparent permeation kinetics and led to different statistical outcomes in the comparative assessment of the two formulations.

The conclusions are restricted to this internally controlled comparison of the two formulations and barrier models tested. Neither model is proposed as a surrogate for excised human skin, and no inference regarding *in vivo* performance is made.

These findings have several practical implications for the development and evaluation of topical formulations. First, because the ordering of the mean flux values for two nominally dose-equivalent commercial gels differed between the barrier models, barrier selection is not merely a methodological consideration; it may also influence the conclusions drawn from *in vitro* permeation studies. Comparative results obtained using a single barrier should therefore be interpreted with appropriate caution. Second, under the present experimental conditions, the standardized synthetic Strat-M® membrane resolved a statistically significant difference in apparent steady-state flux between the two nominally dose-equivalent gels, supporting its potential utility as an animal-free format for early comparative formulation screening. Finally, recognizing barrier-model dependence during early formulation development may reduce the risk of advancing or rejecting formulations based on model-specific behavior, thereby supporting more informed pharmaceutical decision-making. These implications relate to *in vitro* methodology and early formulation development and should not be interpreted as evidence of therapeutic equivalence, bioequivalence, or comparative clinical performance of the two commercial products.

To build on these preliminary findings, future studies should focus on four key areas. First, the HaCaT construct should be quantitatively and functionally characterized using cross-sectional histology with thickness measurements, barrier-integrity testing before and after mounting (e.g., TEER and/or a validated tracer-leakage assay) with predefined acceptance criteria, and independent culture batches to assess inter-batch reproducibility. Second, complete mass-balance recovery should be performed. Third, UV–Vis spectrophotometry should be replaced with a separation-based chromatographic method, such as HPLC or UPLC, to minimize potential interference from residual excipients. Fourth, both barrier systems should be benchmarked in parallel against an established reconstructed human epidermis (EpiDerm™) and against excised human or porcine skin, in a prospectively balanced design using both formulations, independent HaCaT culture batches, and EpiDerm™ tissue lots, and predefined model-appropriate quality and integrity criteria.

Overall, this exploratory study provides a basis for the rational selection and further validation of barrier models in topical formulation development, and a caution against inferring barrier competence from morphological characterization alone.

## Data Availability

The original contributions presented in the study are included in the article/Supplementary Material, further inquiries can be directed to the corresponding author.

## Nomenclature

A: Absorbance; also diffusion area, depending on context
ALI: Air–liquid interface
BSA: Bovine serum albumin
CAS: Chemical Abstracts Service
Cd: Donor concentration
**CO**_**2**_: Carbon dioxide
CPE: Chemical penetration enhancer
CV: Coefficient of variation
DAPI: 4', 6-diamidino-2-phenylindole
EHS: Excised human skin
FBS: Fetal bovine serum
HaCaT: Immortalized human keratinocyte cell line
HPLC: High-performance liquid chromatography
ICH: International Council for harmonization of technical requirements for pharmaceuticals for human use
IgG: Immunoglobulin G
IVIVC: *In vitro–in vivo* correlation
IVPT: *In vitro* permeation testing
JASP: Jeffreys’s Amazing statistics Program
Jss: Steady-state flux
Kow: Octanol–water partition coefficient
Kp: Permeability coefficient
LOD: Limit of detection
LOQ: Limit of quantification
Mw: Molecular weight
n: Number of replicates
NSAIDs: Nonsteroidal anti-inflammatory drugs
OECD: Organization for Economic Co-operation and development
PBS: Phosphate–buffered saline
PET: Polyethylene terephthalate
pKa: Acid dissociation constant
Q: Cumulative permeated mass
σ: Residual standard deviation
Q/A: Cumulative permeated mass per unit area
**R** ^**2**^: Coefficient of determination
RHE: Reconstructed human epidermis
RSD: Relative standard deviation
S: Slope of the calibration curve
SD: Standard deviation
SmPC: Summary of product characteristics
t: Time
TEER: Transepithelial electrical resistance
TEWL: Transepidermal water loss
TG: Test Guideline
*T*lag: Lag time
TLC: Thin-layer chromatography
UPLC: Ultra-performance liquid chromatography
USA: United States of America
UV – Vis: Ultraviolet–visible spectrophotometry
w/w: Weight per weight
λmax: Wavelength of maximum absorbance
