# Supplementary material for: Barrier model selection alters apparent diclofenac permeation parameters: a controlled in vitro comparison of Strat-M® and a HaCaT-based epidermal construct
Source: Front Pharmacol. 2026 Oct 7;17:1934405. doi: 10.3389/fphar.2026.1934405 (PMC13684325; doi:10.3389/fphar.2026.1934405)
Supplement: Supplementary file 1 [file Supplementaryfile1.docx]

Supplementary Material

# 1 Supplementary Figures and Tables

## 1.1 Supplementary Tables

| **Parameter** | **Strat-M®** | **HaCaT construct** | **EpiDerm™ (RHE, primary keratinocytes)** | **Native human epidermis/skin (reference)** |
| --- | --- | --- | --- | --- |
| ***Model identity and architecture*** | | | | |
| **1. Model type, origin, cell source, and presence of viable cells** | Synthetic multilayer membrane; polyethersulfone layers over a polyolefin support, impregnated with a proprietary lipid blend. No cells, no metabolic or repair capacity.ᵃ,ᶜ (17–19) | Epidermal equivalent reconstructed from the spontaneously immortalized, aneuploid human keratinocyte line HaCaT. Viable cells present.ᵇ (20,21) | Commercial reconstructed human epidermis from primary human keratinocytes cultured at the air–liquid interface; viable cells present. EpiDerm-200-X was used in the FDA parallel evaluation.ᵃ,ᵇ (22–25) | Excised human skin or heat-separated human epidermis; viability depends on preparation and storage. Reference barrier.ᵇ,ᶜ (5,24) |
| **2. Total thickness and rate-limiting barrier layer (µm)** | Total thickness ≈300–325 µm. Resistance arises from the tight uppermost polyethersulfone layer and lipid impregnation; the diffusion path is a tortuous synthetic matrix rather than ordered intercellular lipid lamellae.ᵃ,ᵇ (18,19,26) | Not reported for the present construct. An independent HaCaT-based epidermal equivalent showed a stratum corneum described as modest (11); a separate comparison found HaCaT-derived epidermis thinner than that from primary keratinocytes and devoid of stratum granulosum and corneum, so no defined rate-limiting corneal layer.ᵇ,† (11,21) | Total tissue thickness 70–130 µm (mean 111.0 µm), with at least four viable cell layers (EPI-200 lot histology); supplier specification 8–12 cell layers plus stratum corneum. The rate-limiting barrier is the stratum corneum.ᵃ,ᵇ (22) | Stratum corneum 18.3 ± 4.9 µm (dorsal forearm), 11.0 ± 2.2 µm (shoulder), 14.9 ± 3.4 µm (buttock); viable epidermis 56.6 ± 11.5, 70.3 ± 13.6, and 81.5 ± 15.7 µm, respectively (n = 71 donors). The rate-limiting barrier is the stratum corneum.ᵇ (19,28,29) |
| **3. Stratum corneum: presence, continuity, and corneocyte layer number** | No true stratum corneum. A tight surface layer and lipid coating simulate stratum corneum function; corneocytes are absent. Corneocyte layer number not applicable.ᵃ,ᵇ (17,26) | Discrepant between laboratories. Two independent reports describe HaCaT-derived epidermis as devoid of stratum granulosum and corneum, or a stratum corneum as lacking (21,30); a third reports a stratum corneum that is present but modest and irregular (11). Corneocyte layer number not reported. The discrepancy indicates strong protocol dependence rather than a single characteristic value.ᵇ,† (11,21,30) | Characteristic stratum corneum present; extracellular lipid lamellae observed, but not regular throughout the full stratum corneum. Corneocyte layer number not reported.ᵃ,ᵇ (22–24) | True stratum corneum present. The number of corneocyte layers is strongly site-dependent, ranging from approximately 50 layers in palmoplantar skin to fewer than 10 in facial and genital skin; corneocytes are approximately 30 µm in diameter and 0.5–0.8 µm thick.ᵇ (29,31,32) |
| ***Barrier composition and function*** | | | | |
| **4. Stratum corneum lipid composition and lamellar organization (class level)** | Proprietary synthetic lipid blend applied in ratios intended to resemble human stratum corneum; pores are lipid-impermeabilized. Organization is synthetic rather than biological lamellae; ceramide, free fatty acid, and cholesterol profiles are not reported.ᵃ,ᶜ (17,19) | Impaired barrier-lipid synthesis reported for HaCaT constructs: free fatty acids absent, ceramides very low and incomplete, and abnormal stratum corneum lipid organization. No quantitative lipid profiling of HaCaT epidermal equivalents was identified. Not measured in the present construct.ᶜ,† (20,33) | All major epidermal lipid classes are present. Relative to native epidermis, polar ceramides 5 and 6 are lower, ceramide 7 is absent, and free fatty acids are very low; the long periodicity phase is present, but the short periodicity phase is missing.ᵇ (23,34) | Ceramides, cholesterol, and free fatty acids are arranged in organized stacked bilayers/lamellae; the composition most closely matching this arrangement best reproduces permeability behavior. (29) |
| **5. Epidermal differentiation status and organization** | Not applicable — no viable cells and no epidermal differentiation programme.ᵃ (17) | Abnormal and incomplete terminal differentiation. K1/K10 expression irregular, K6/K16 present in all layers; transcription of cornified-envelope proteins (filaggrin, loricrin, involucrin) abnormal relative to primary keratinocytes; karyotype aneuploid. One HaCaT-based equivalent expressed CK10, CK14, involucrin, and filaggrin, but only at a modest level in the stratum corneum. Differentiated HaCaT cells can upregulate KRT1, KRT10, and IVL under some conditions, but three-dimensional barrier maturation remains weak.ᵇ,† (11,20,21,30,33) | Completely stratified epithelium closely resembling normal human epidermis, although differentiation-marker expression is aberrant for K6, involucrin, and transglutaminase.ᵇ (34) | Normal terminal differentiation generates the stratum corneum from keratinocytes; native structure is the benchmark for organization and barrier maturation.ᵇ (29) |
| **6. Barrier integrity metrics: TEER, TEWL** | Not applicable — TEER and TEWL are viable-tissue integrity readouts. Not reported for Strat-M®, which is used as a permeation membrane rather than a barrier-readout system.ᵇ,ᵈ (18) | HaCaT monolayers did not form a robust barrier: no increase in TEER followed calcium-induced differentiation, in contrast to primary human foreskin keratinocytes and N/TERT2G cells, both of which increased. In three-dimensional comparisons, TEER was lower than for better-performing immortalized lines. Absolute TEER values not reported.ᵇ,† (21,35) | TEWL was elevated relative to excised human skin for EpiDerm-200-X; absolute values were reported graphically only. Selected in earlier validation work as the RHE with the best barrier properties among those tested.ᵇ (24,25) | Pooled TEWL reference values for healthy adults range from 2.3 g m⁻² h⁻¹ (95% CI 1.9–2.7; breast) to 44.0 g m⁻² h⁻¹ (95% CI 39.8–48.2; axilla) across 86 skin areas in 212 studies. IVPT integrity acceptance thresholds are laboratory- and guideline-dependent (e.g., <10 or <15 g m⁻² h⁻¹) rather than universal.ᵇ,ᶜ (5,24,36) |
| **7. Reported permeation behavior, probe compounds, and correlation with human skin** | Good correlation with human cadaver skin for nicotine formulations; closer to human skin than EpiSkin® for resorcinol flux; diclofenac formulation trends correlated with human skin equivalent; amphotericin B R² = 0.99 and f2 = 85; rivastigmine profile similar to pig skin (R² = 0.920) with IVIVC R² = 0.991, though lower than human skin in later work. In the FDA parallel evaluation, the lowest permeation was observed for all three probes. Permeability coefficients not reported.ᵇ (10,17,18,24,37–39) | Lucifer yellow was not retained and passed completely through HaCaT-derived epidermis, whereas other immortalized lines excluded the dye comparably to primary keratinocytes, indicating poor barrier function. No permeability coefficient or drug permeation data identified .ᵇ,† (21) | 6 h cumulative permeation of a finite dose (6 nmol cm⁻²) was highest in EpiDerm-200-X for caffeine and testosterone (EpiDerm > EHS > Strat-M®), whereas salicylic acid permeated most in EHS (EHS > EpiDerm > Strat-M®). RHE permeation generally exceeds that of human epidermis, but the substance ranking reflects this. Permeability coefficients not reported.ᵇ (24,25) | Reference standard. 6 h cumulative permeation in EHS: caffeine 0.97 ± 0.22, testosterone 0.33 ± 0.13, and salicylic acid 0.18 ± 0.10 nmol cm⁻² (log Kow −0.07, 3.32, and 2.26, respectively). Human skin equivalent discriminated caffeine from testosterone, whereas Episkin did not; for the rivastigmine IVIVC, human skin yielded the highest correlation coefficient among the membranes tested.ᵇ (24,39,40) |
| **8. Known sources of variability** | Low batch-to-batch variation, straightforward storage and handling, reproducible performance. Compound- and formulation-dependent deviations from human skin nonetheless persist.ᵃ,ᵇ (18,26,37) | Donor variation is avoided because the line is immortalized, but architecture and differentiation depend strongly on the protocol —calcium concentration, culture duration, time at the air–liquid interface, and passage number. Reported stratum corneum status ranges from absent to modest and irregular between laboratories, so no single representative value can be given.ᵇ,† (11,20,21,30) | Low intra-batch variation, but moderate to considerable inter-batch variation in tissue architecture and marker expression; reproducibility not clearly superior to excised skin.ᵇ (25,34) | Varies with donor, anatomical site, collection, preparation, handling, and storage; intra- and inter-lot variability of up to 37% and 50%, respectively, has been reported.ᶜ (5,19) |
| ***Application to in vitro permeation testing*** | | | | |
| **9. Demonstrated formulation-discrimination capability** | Demonstrated for nicotine/CPE formulations, diclofenac hydrogels versus creams, rivastigmine transdermal patches across membranes, and dissolving microarray patches.ᵇ (17,18,26,39) | Not demonstrated. The available literature addresses cell biology and barrier weakness rather than comparative formulation discrimination.ᵇ,ᵈ,† (20,21) | Supports permeation ranking across substances and formulations; selected for finite-dose work as the RHE with the best barrier properties among those tested.ᵇ (24,25) | The comparator for formulation discrimination and for regulatory IVPT and bioequivalence work.ᶜ (5,24) |
| **10. Reproducibility, availability, and regulatory/ethical status** | Commercial, non-animal, readily available, long shelf life, and reproducible; useful for reducing the use of human and animal tissue. No regulatory validation as a skin-permeation surrogate.ᵃ,ᵇ (7,18,37,38) | Readily cultured, immortalized line with no donor constraints and low cost. Not validated for any regulatory purpose and not established as a permeability surrogate.ᶜ,ᵈ (20,21) | Commercially available. RHE models are regulatory-validated for skin irritation (OECD TG 439) and skin corrosion (OECD TG 431). Not validated for dermal absorption: OECD TG 428 specifies the use of excised human or animal skin. Reproducibility is not markedly better than that of excised skin.ᵃ,ᶜ (6,16,25,41) | Limited supply, high cost, ethical and logistical constraints, and an integrity-control burden. Remains the accepted reference standard under OECD TG 428 and IVPT guidance.ᶜ (5,6,7) |
| **11. Principal limitation and appropriate scope of use** | Absence of viable biology and a tortuous synthetic diffusion path rather than ordered stratum corneum lipid lamellae, so absolute prediction is imperfect. Role: standardized comparative screening and formulation ranking under defined conditions, and reduction of biological membrane use, prior to confirmatory testing.ᵇ (17,26,38) | Incomplete terminal differentiation and absent or, at best, modest and irregular stratum corneum formation, providing no adequate physical barrier for quantitative permeation. Role: standardized comparative ranking where a viable, stratified barrier is required; not a replacement for human skin where absolute permeability matters.ᵇ,† (11,21,30) | Higher permeability than native epidermis and non-native lipid organization; validated for irritation and corrosion but not for absorption. Role: standardized comparative ranking where a viable, stratified barrier is required; not a replacement for human skin where absolute permeability matters.ᵇ (23–25) | Limitation: supply, cost, donor and preparation variability, and integrity-control burden. Role: final reference standard for confirmatory IVPT and bioequivalence-relevant testing.ᶜ (5,24,42) |

**Table 1.** Comparative characterization of the two barrier models used in this study (Strat-M® and a HaCaT-based epidermal equivalent) against a reconstructed human epidermis model (EpiDerm™) and native human epidermis, compiled from published characterization studies and manufacturer documentation (5–7,10,11,16–42).

**Provenance of data**

ᵃ Manufacturer documentation. ᵇ Experimentally measured in a peer-reviewed study. ᶜ Reported in the literature without primary measurement. ᵈ Not reported.

† Reported for HaCaT-derived epidermal equivalents in the cited studies; not measured in the present construct. The construct used here was not characterized for barrier function, lipid composition, or stratum corneum architecture, and no property in this column should be read as a measured property of it.

**Notes**

“Not reported” indicates that the parameter was not reported in the sources consulted; “not applicable” indicates that the parameter has no meaningful value for that model. No value has been inferred from a related model.

Numerical values are given as reported ranges or as mean ± SD, with sample size consistently reported. Units are stated in every case.

Permeability coefficients (Kp, cm h⁻¹) were not reported for any of the four models in the sources consulted; row 7 therefore summarises reported permeation behavior, probe compounds, and correlation statistics rather than harmonized coefficients.

Where published findings for HaCaT constructs conflict (rows 2, 3, and 8), both are presented rather than reconciled, since the divergence reflects genuine protocol dependence of stratum corneum formation in this class of model.

**Abbreviations:** CPE, chemical penetration enhancer; EHS, excised human skin; IVIVC, *in vitro–in vivo* correlation; IVPT, *in vitro* permeation testing; Kow, octanol–water partition coefficient; Kp, permeability coefficient; RHE, reconstructed human epidermis; TEER, transepithelial/transepidermal electrical resistance; TEWL, transepidermal water loss.

**References cited in Table 1 (numbering follows the manuscript reference list)**

5. Lane ME. In vitro permeation testing for the evaluation of drug delivery to the skin. Eur J Pharm Sci. 2024 Oct 1;201:106873. doi:10.1016/j.ejps.2024.106873

6. OECD. Test No. 428: Skin Absorption: In Vitro Method. OECD Guidel Test Chem Sect 4. 2004 Nov 22. doi:10.1787/9789264071087-en

7. Neupane R, Boddu SHS, Renukuntla J, Babu RJ, Tiwari AK. Alternatives to Biological Skin in Permeation Studies: Current Trends and Possibilities. Pharmaceutics. 2020 Feb 13;12(2):152. doi:10.3390/pharmaceutics12020152 PubMed PMID: 32070011; PubMed Central PMCID: PMC7076422.

10. Kichou H, Bonnier F, Dancik Y, Bakar J, Michael-Jubeli R, Caritá AC, et al. Strat-M® positioning for skin permeation studies: A comparative study including EpiSkin® RHE, and human skin. Int J Pharm. 2023 Nov 25;647:123488. doi:10.1016/j.ijpharm.2023.123488 PubMed PMID: 37805151.

11. Mini CA, Dreossi S a. C, Abe FR, Maria-Engler SS, Oliveira DP. Immortalized keratinocytes cells generates an effective model of Epidermal Human Equivalent for irritation and corrosion tests. Toxicol Vitro Int J Publ Assoc BIBRA. 2021 Mar;71:105069. doi:10.1016/j.tiv.2020.105069 PubMed PMID: 33309870.

16. OECD. Test No. 439: In Vitro Skin Irritation: Reconstructed Human Epidermis Test Method. OECD Guidel Test Chem Sect 4. 2025 Jun 24. doi:10.1787/9789264242845-en

17. Haq A, Goodyear B, Ameen D, Joshi V, Michniak-Kohn B. Strat-M® synthetic membrane: Permeability comparison to human cadaver skin. Int J Pharm. 2018 Aug 25;547(1):432–7. doi:10.1016/j.ijpharm.2018.06.012

18. Kovács A, Zsikó S, Falusi F, Csányi E, Budai-Szűcs M, Csóka I, et al. Comparison of Synthetic Membranes to Heat-Separated Human Epidermis in Skin Permeation Studies In Vitro. Pharmaceutics. 2021 Dec;13(12):2106. doi:10.3390/pharmaceutics13122106

19. Magnano GC, Sut S, Dall’Acqua S, Di Cagno MP, Lee L, Lee M, et al. Validation and testing of a new artificial biomimetic barrier for estimation of transdermal drug absorption. Int J Pharm. 2022 Nov 25;628:122266. doi:10.1016/j.ijpharm.2022.122266 PubMed PMID: 36228883.

20. Boelsma E, Verhoeven MC, Ponec M. Reconstruction of a human skin equivalent using a spontaneously transformed keratinocyte cell line (HaCaT). J Invest Dermatol. 1999 Apr;112(4):489–98. doi:10.1046/j.1523-1747.1999.00545.x PubMed PMID: 10201534.

21. Jahn M, Lang V, Diehl S, Back R, Kaufmann R, Fauth T, et al. Different immortalized keratinocyte cell lines display distinct capabilities to differentiate and reconstitute an epidermis in vitro. Exp Dermatol. 2024;33(1):e14985. doi:10.1111/exd.14985

22. EpiDerm in vitro 3D Tissue. Mattek - Part of Sartorius (Internet). (cited 2026 May 15). Available from: https://www.mattek.com/mattek-product/epiderm/

23. Ponec M, Boelsma E, Weerheim A, Mulder A, Bouwstra J, Mommaas M. Lipid and ultrastructural characterization of reconstructed skin models. Int J Pharm. 2000 Aug 10;203(1–2):211–25. doi:10.1016/s0378-5173(00)00459-2 PubMed PMID: 10967443.

24. Salminen AT, Davis KJ, Felton RP, Nischal N, VonTungeln LS, Beland FA, et al. Parallel evaluation of alternative skin barrier models and excised human skin for dermal absorption studies in vitro. Toxicol Vitro Int J Publ Assoc BIBRA. 2023 Sep;91:105630. doi:10.1016/j.tiv.2023.105630 PubMed PMID: 37315744; PubMed Central PMCID: PMC10527924.

25. Schäfer-Korting M, Bock U, Diembeck W, Düsing HJ, Gamer A, Haltner-Ukomadu E, et al. The Use of Reconstructed Human Epidermis for Skin Absorption Testing: Results of the Validation Study. Altern Lab Anim. 2008 May 1;36(2):161–87. doi:10.1177/026119290803600207

26. Anjani QK, Nainggolan ADC, Li H, Miatmoko A, Larrañeta E, Donnelly RF. Parafilm® M and Strat-M® as skin simulants in in vitro permeation of dissolving microarray patches loaded with proteins. Int J Pharm. 2024 Apr 25;655:124071. doi:10.1016/j.ijpharm.2024.124071 PubMed PMID: 38554738.

27. Kocsis D, Klang V, Schweiger E, Varga-Medveczky Z, Mihály A, Pongor C, et al. Characterization and ex vivo evaluation of excised skin samples as substitutes for human dermal barrier in pharmaceutical and dermatological studies. Skin Res Technol. 2022 Jun 21;28(5):664–76. doi:10.1111/srt.13165 PubMed PMID: 35726964; PubMed Central PMCID: PMC9907592.

28. Sandby-Møller J, Poulsen T, Wulf HC. Epidermal thickness at different body sites: relationship to age, gender, pigmentation, blood content, skin type and smoking habits. Acta Derm Venereol. 2003;83(6):410–3. doi:10.1080/00015550310015419 PubMed PMID: 14690333.

29. Lundborg M, Narangifard A, Wennberg CL, Lindahl E, Daneholt B, Norlén L. Human skin barrier structure and function analyzed by cryo-EM and molecular dynamics simulation. J Struct Biol. 2018 Aug;203(2):149–61. doi:10.1016/j.jsb.2018.04.005 PubMed PMID: 29702212.

30. Smits JPH, Niehues H, Rikken G, van Vlijmen-Willems IMJJ, van de Zande GWHJF, Zeeuwen PLJM, et al. Immortalized N/TERT keratinocytes as an alternative cell source in 3D human epidermal models. Sci Rep. 2017 Sep 19;7(1):11838. doi:10.1038/s41598-017-12041-y PubMed PMID: 28928444; PubMed Central PMCID: PMC5605545.

31. Ya-Xian Z, Suetake T, Tagami H. Number of cell layers of the stratum corneum in normal skin - relationship to the anatomical location on the body, age, sex and physical parameters. Arch Dermatol Res. 1999 Oct;291(10):555–9. doi:10.1007/s004030050453 PubMed PMID: 10552214.

32. Holbrook KA, Odland GF. Regional differences in the thickness (cell layers) of the human stratum corneum: an ultrastructural analysis. J Invest Dermatol. 1974 Apr;62(4):415–22. doi:10.1111/1523-1747.ep12701670 PubMed PMID: 4820685.

33. Kim JE, Kim WH, Kim S, Na Y, Choi J, Hong YD, et al. Bioconversion of retinol and its cell barrier function in human immortalized keratinocytes cells and artificial epidermis-dermis skin. Exp Dermatol. 2023 Jun;32(6):822–30. doi:10.1111/exd.14781 PubMed PMID: 36843342.

34. Ponec M, Boelsma E, Gibbs S, Mommaas M. Characterization of reconstructed skin models. Skin Pharmacol Appl Skin Physiol. 2002;15 Suppl 1:4–17. doi:10.1159/000066682 PubMed PMID: 12476005.

35. Moran MC, Pandya RP, Leffler KA, Yoshida T, Beck LA, Brewer MG. Characterization of Human Keratinocyte Cell Lines for Barrier Studies. JID Innov Skin Sci Mol Popul Health. 2021 Jun;1(2):100018. doi:10.1016/j.xjidi.2021.100018 PubMed PMID: 34909717; PubMed Central PMCID: PMC8659750.

36. Akdeniz M, Gabriel S, Lichterfeld-Kottner A, Blume-Peytavi U, Kottner J. Transepidermal water loss in healthy adults: a systematic review and meta-analysis update. Br J Dermatol. 2018 Nov;179(5):1049–55. doi:10.1111/bjd.17025 PubMed PMID: 30022486.

37. Kaur L, Singh K, Paul S, Singh S, Singh S, Jain SK. A Mechanistic Study to Determine the Structural Similarities Between Artificial Membrane Strat-MTM and Biological Membranes and Its Application to Carry Out Skin Permeation Study of Amphotericin B Nanoformulations. AAPS PharmSciTech. 2018 May;19(4):1606–24. doi:10.1208/s12249-018-0959-6 PubMed PMID: 29488196.

38. Simon A, Amaro MI, Healy AM, Cabral LM, de Sousa VP. Comparative evaluation of rivastigmine permeation from a transdermal system in the Franz cell using synthetic membranes and pig ear skin with in vivo-in vitro correlation. Int J Pharm. 2016 Oct 15;512(1):234–41. doi:10.1016/j.ijpharm.2016.08.052 PubMed PMID: 27568498.

39. Ribeiro VO, Nader Mota GC, Moreira LS, Alcântara Guimarães AL de, Macêdo Vieira AC de, Sousa VP de, et al. In vitro–in vivo correlation for consolidation of permeation parameters using different membranes in Franz Cell with a transdermal rivastigmine patch. J Drug Deliv Sci Technol. 2026 Jun 1;120:108225. doi:10.1016/j.jddst.2026.108225

40. Netzlaff F, Kaca M, Bock U, Haltner-Ukomadu E, Meiers P, Lehr CM, et al. Permeability of the reconstructed human epidermis model Episkin in comparison to various human skin preparations. Eur J Pharm Biopharm Off J Arbeitsgemeinschaft Pharm Verfahrenstechnik EV. 2007 Apr;66(1):127–34. doi:10.1016/j.ejpb.2006.08.012 PubMed PMID: 17029766.

41. OECD. Test No. 431: In vitro skin corrosion: reconstructed human epidermis (RHE) test method. OECD Guidel Test Chem Sect 4. 2025 Jun 24. doi:10.1787/9789264264618-en

42. Marques M, Teixeira F, Vieira M, Amaral MH, Costa PC, Dall’Acqua S, et al. Unravelling polyphenol skin permeation: A comparative study using full-thickness 3D in vitro and ex vivo human skin models. Int J Cosmet Sci. n/a(n/a). doi:10.1111/ics.70107

| Component | Voltaren ® Emulgel 11.6 mg/g gel | Olfen ® 10 mg/g gel |
| --- | --- | --- |
| Active substance / salt / conc. | Diclofenac diethylammonium / 11.6 mg/g (equivalent to 10 mg/g diclofenac sodium) | Diclofenac sodium / 10 mg/g |
| Vehicle / co-solvents | Purified water, isopropyl alcohol, propylene glycol (E1520) | Purified water, isopropyl alcohol |
| Gelling agent | Carbomer | Hydroxyethyl cellulose, hydroxypropyl cellulose |
| Formulation type | Emulgel, consisting of a biphasic oil-in-water system incorporated into a gel matrix | Simple hydrogel formulation |
| Permeation enhancers (recognised) | Propylene glycol, isopropyl alcohol, cocoyl caprylocaprate, macrogol cetostearyl ether | Diisopropyl adipate, isopropyl alcohol |
| Other notable excipients | Diethylamine, liquid paraffin, perfume cream 45 (contains allergens: benzyl alcohol, benzyl benzoate, etc.) | Sodium metabisulfite (E223), lactic acid |

**Table 2.** Composition of the two commercial diclofenac gels evaluated in the present study, compiled from manufacturers' SmPCs and packaging inserts (43,44).

References cited in Table 2 (numbering follows the manuscript reference list)

- Centralna baza zdravil 2 - Zdravilo: Voltaren Emulgel 11,6 mg/g gel 50 g [Internet]. [cited 2026 May 4]. Available from: <https://www.cbz.si/cbz/bazazdr2.nsf/o/F9844FD95C67EA54C12579C2003F6977?opendocument>
- Centralna baza zdravil 2 - Zdravilo: Olfen 10 mg/g gel 50 g [Internet]. [cited 2026 May 4]. Available from: <https://www.cbz.si/cbz/bazazdr2.nsf/o/CEA7CC347E4B3560C12579C2003F5908?opendocument>

| Barrier model | Formulation | Jss (µgcm⁻²h⁻¹) | *t*lag (h) | Kp × 10⁻³ (cmh⁻¹) |
| --- | --- | --- | --- | --- |
| HaCaT  Strat-M® | Voltaren®  Olfen®  Voltaren®  Olfen® | 66.35 ± 8.08  62.20 ± 8.11  48.03 ± 10.38 *  85.30 ± 14.90 * | 0.49 ± 0.31 #  0.24 ± 0.08 #  2.23 ± 0.69 #  1.21 ± 0.39 # | 6.63 ± 0.81  6.22 ± 0.81  4.80 ± 1.04  8.53 ± 1.49 |

(*) Statistically significant difference (p < 0.05) between formulations within the same barrier model. On Strat-M®, Jss was approximately 1.8-fold higher for Olfen® than for Voltaren® (mean difference 37.3 µgcm⁻²h⁻¹, 95% CI 6.72 to 67.81; p = 0.029), whereas the two formulations did not differ significantly in Jss on HaCaT (p = 0.565).

(#) Statistically significant difference (p < 0.05) between barrier models for the same formulation. *T*lag was significantly longer on Strat-M® than on HaCaT for both Voltaren® (p = 0.033) and Olfen® (p = 0.046). In contrast, between-model differences in Jss did not reach significance for either formulation (Voltaren®: p = 0.077; Olfen®: p = 0.097), and *t*lag did not differ significantly between the two formulations within either barrier model.

**Table 3.** Permeation parameters for Voltaren® and Olfen® gels across the HaCaT and Strat-M® barrier models. Data are expressed as mean ± SD (n = 3 technical replicates per group). Comparisons were performed using independent samples Welch's t-tests, with statistical significance set at p < 0.05.

## 1.2 Supplementary Material

| **Formulation/model** | **T*lag* – zero substitution (h)** | **T*lag* – LOQ/2 substitution (h)** | **T*lag* – LOQ/√2 substitution (h)** | **T*lag* – LOQ substitution (h)** |
| --- | --- | --- | --- | --- |
| Voltaren® / HaCaT | 0.49 ± 0.31 | 0.45 ± 0.36 | 0.43 ± 0.38 | 0.40 ± 0.42 |
| Olfen® / HaCaT | 0.24 ± 0.08 | 0.24 ± 0.08 | 0.24 ± 0.08 | 0.24 ± 0.08 |
| Voltaren® / Strat-M® | 2.23 ± 0.69 | 1.75 ± 0.55 | 1.55 ± 0.50 | 1.27 ± 0.44 |
| Olfen® / Strat-M® | 1.21 ± 0.39 | 1.06 ± 0.43 | 1.00 ± 0.44 | 0.92 ± 0.47 |
| Strat-M® / HaCaT ratio, Voltaren® | 4.5× | 3.9× | 3.6× | 3.2× |
| Strat-M® / HaCaT ratio, Olfen® | 5.0× | 4.4× | 4.2× | 3.8× |

**Table S1.** Sensitivity of *t*lag to alternative numerical treatments of receptor concentrations below the limit of quantification (LOQ). Values are mean ± SD (n = 3). Substitution values of 0, LOQ/2, LOQ/√2, and LOQ were evaluated to examine the sensitivity of the estimated *t*lag to different plausible treatments of below-LOQ observations. Zero substitution is the approach used throughout the manuscript. Jss and Kp were unaffected by the substitution and are therefore not shown. This analysis is intended to assess the sensitivity of the estimated *t*lag to the handling of below-LOQ data, rather than to identify a preferred substitution method.

| **Model** | **Formulation** | **Replicate** | **Regression window (min)** | **n** | **Jss (µg cm⁻² h⁻¹)** | ***T*lag (min)** | **R^2^** |
| --- | --- | --- | --- | --- | --- | --- | --- |
| HaCaT | Voltaren® | A | 60–240 | 4 | 74.380382 | 51.013795 | 0.9961 |
| HaCaT | Voltaren® | B | 60–240 | 4 | 66.442955 | 18.778653 | 0.9953 |
| HaCaT | Voltaren® | C | 120–300 | 4 | 58.213692 | 19.103226 | 0.9755 |
| HaCaT | Olfen® | A | 30–180 | 4 | 71.267455 | 14.694884 | 0.9907 |
| HaCaT | Olfen® | B | 30–240 | 5 | 59.722951 | 18.683830 | 0.9887 |
| HaCaT | Olfen® | C | 30–240 | 5 | 55.617452 | 9.682149 | 0.9791 |
| Strat-M® | Voltaren® | A | 120–300 | 4 | 59.822696 | 113.432987 | 0.9709 |
| Strat-M® | Voltaren® | B | 120–360 | 5 | 40.243168 | 106.302934 | 0.9934 |
| Strat-M® | Voltaren® | C | 180–300 | 3 | 44.033228 | 181.430000 | 0.9413 |
| Strat-M® | Olfen® | A | 120–240 | 3 | 99.113947 | 98.813487 | 0.9946 |
| Strat-M® | Olfen® | B | 60–300 | 5 | 69.507238 | 53.806721 | 0.9885 |
| Strat-M® | Olfen® | C | 60–240 | 4 | 87.266900 | 64.661842 | 0.9834 |

**Table S2**. Linear regression time windows and permeation parameters (Jss, *t*lag, R^2^) by formulation and barrier model.

The table summarizes the regression windows selected for each individual diffusion-cell replicate, together with the number of included data points, the previously calculated steady-state flux (Jss), lag time (*t*lag), and the corresponding coefficient of determination ($R^{2}$). The $R^{2}$values indicate the goodness of fit of the linear regression within the selected time interval.

| Comparison | Parameter | Mean difference (95%  CI) | (p) | Hedges’ $g$ (95% CI) |
| --- | --- | --- | --- | --- |
| HaCaT: Olfen® − Voltaren® | Jss | −4.143 (−22.503, 14.217) | 0.565 | −0.408 (−2.008, 1.239) |
|  | *t*lag | −15.278 (−58.136, 27.580) | 0.287 | −0.905 (−2.615, 0.949) |
| Strat-M®: Olfen® − Voltaren® | Jss | 37.263 (6.719, 67.807) | 0.029 | 2.315 (−0.032, 4.531) |
|  | *t*lag | −61.294 (−146.356, 23.768) | 0.108 | −1.451 (-3.312, 0.549) |
| Voltaren®: HaCaT − Strat-M® | Jss | 18.313 (−3.293, 39.918) | 0.077 | 1.570 (−0.424, 3.435) |
|  | *t*lag | −104.089 (−191.652, −16.527) | 0.033 | −2.586 (−5.115, 0.058) |
| Olfen®: HaCaT − Strat-M® | Jss | −23.093 (−53.761, 7.574) | 0.097 | −1.536 (−3.438, 0.509) |
|  | *t*lag | −58.074 (−113.744, −2.403) | 0.046 | −2.740 (−5.582, 0.176) |

**Table S3.** Pairwise statistical comparisons of permeation parameters.

Pairwise comparisons were performed using two-sided Welch’s independent-samples $t$-tests. Mean differences and Hedges’ $g$effect sizes were calculated as the first-listed group minus the second-listed group and are presented with two-sided 95% confidence intervals. Given the small sample size, statistical comparisons were interpreted as exploratory. Statistical significance was set at $p<0.05$. $K_{p}$was calculated directly from $J_{\mathrm{ss}}/C_{d}$and was therefore not tested separately.
